# Water T_2_ as an early, global and practical biomarker for metabolic syndrome: an observational cross-sectional study

**DOI:** 10.1186/s12967-017-1359-5

**Published:** 2017-12-19

**Authors:** Michelle D. Robinson, Ina Mishra, Sneha Deodhar, Vipulkumar Patel, Katrina V. Gordon, Raul Vintimilla, Kim Brown, Leigh Johnson, Sid O’Bryant, David P. Cistola

**Affiliations:** 1 Nanoparticle Diagnostics Laboratory, Institute for Cardiovascular & Metabolic Diseases, University of North Texas Health Science Center, Fort Worth, TX 76107 USA; 2 Center for Alzheimer’s & Neurodegenerative Diseases Research, Institute for Healthy Aging, University of North Texas Health Science Center, Fort Worth, TX 76107 USA; 3 Department of Clinical Laboratory Science, College of Allied Health Sciences, East Carolina University, Greenville, NC 27834 USA; 4 Department of Biochemistry & Molecular Biology, The Brody School of Medicine, East Carolina University, Greenville, NC 27834 USA; 5 The East Carolina Diabetes & Obesity Institute, East Carolina University, Greenville, NC 27834 USA; 6 Center of Emphasis in Diabetes & Metabolism, Paul L. Foster School of Medicine, Texas Tech University Health Sciences Center El Paso, El Paso, TX 79905 USA

**Keywords:** Metabolic syndrome, Insulin resistance, Inflammation, dyslipidemia, magnetic resonance relaxometry, T_2_, transverse relaxation time, type 2 diabetes mellitus, Atherosclerosis, cardiovascular disease

## Abstract

**Background:**

Metabolic syndrome (MetS) is a highly prevalent condition that identifies individuals at risk for type 2 diabetes mellitus and atherosclerotic cardiovascular disease. Prevention of these diseases relies on early detection and intervention in order to preserve pancreatic β-cells and arterial wall integrity. Yet, the clinical criteria for MetS are insensitive to the early-stage insulin resistance, inflammation, cholesterol and clotting factor abnormalities that characterize the progression toward type 2 diabetes and atherosclerosis. Here we report the discovery and initial characterization of an atypical new biomarker that detects these early conditions with just one measurement.

**Methods:**

Water T_2_, measured in a few minutes using benchtop nuclear magnetic resonance relaxometry, is exquisitely sensitive to metabolic shifts in the blood proteome. In an observational cross-sectional study of 72 non-diabetic human subjects, the association of plasma and serum water T_2_ values with over 130 blood biomarkers was analyzed using bivariate, multivariate and logistic regression.

**Results:**

Plasma and serum water T_2_ exhibited strong bivariate correlations with markers of insulin, lipids, inflammation, coagulation and electrolyte balance. After correcting for confounders, low water T_2_ values were independently and additively associated with fasting hyperinsulinemia, dyslipidemia and subclinical inflammation. Plasma water T_2_ exhibited 100% sensitivity and 87% specificity for detecting early insulin resistance in normoglycemic subjects, as defined by the McAuley Index. Sixteen normoglycemic subjects with early metabolic abnormalities (22% of the study population) were identified by low water T_2_ values. Thirteen of the 16 did not meet the harmonized clinical criteria for metabolic syndrome and would have been missed by conventional screening for diabetes risk. Low water T_2_ values were associated with increases in the mean concentrations of 6 of the 16 most abundant acute phase proteins and lipoproteins in plasma.

**Conclusions:**

Water T_2_ detects a constellation of early abnormalities associated with metabolic syndrome, providing a global view of an individual’s metabolic health. It circumvents the pitfalls associated with fasting glucose and hemoglobin A1c and the limitations of the current clinical criteria for metabolic syndrome. Water T_2_ shows promise as an early, global and practical screening tool for the identification of individuals at risk for diabetes and atherosclerosis.

**Electronic supplementary material:**

The online version of this article (10.1186/s12967-017-1359-5) contains supplementary material, which is available to authorized users.

## Background

Metabolic syndrome (MetS) is one of the most prevalent public health problems of the twenty-first century [1–3]. In the US, approximately one-third of adults and half of those ≥ 60 years of age have MetS [2, 3]. Previously called insulin resistance syndrome or syndrome X, MetS can be defined in two ways [4–6]: (i) generally, as a constellation of abnormalities that includes insulin resistance, glucose intolerance, abdominal obesity, elevated blood pressure, dyslipidemia, and/or a pro-inflammatory, pro-thrombotic state, and (ii) specifically, as a set of clinical criteria and cutoffs. The definitions and criteria for MetS have been the subject of considerable debate [4–9]. The Cardiometabolic Think Tank was convened in 2014 in an attempt to develop a consensus on affirmed and emerging concepts as well as recommendations regarding MetS [5]. The concepts are sound, but questions remain about how best to capture the heterogeneity of MetS, particularly the subtypes, stages and unmeasured or residual risk factors [5].

The primary importance of MetS is in identifying individuals at increased risk for type 2 diabetes and atherosclerotic cardiovascular disease [5, 6, 10–14]. The prevention of these diseases hinges on early detection and intervention in order to preserve pancreatic β-cell function and the integrity of the arterial wall [15, 16]. Yet, the clinical criteria and cutoffs for MetS appear to be insensitive to the early-stage metabolic abnormalities that put individuals at risk.

In the progression toward type 2 diabetes, the hallmark early abnormality is insulin resistance [17]. Because of the compensatory hypersecretion of insulin by intact β-cells, fasting glucose levels remain in the normal range during early-stage insulin resistance [17–22]. By the time an individual develops impaired fasting glucose (≥ 100 mg/dL)—one criterion for MetS—a significant decline in β-cell function has already occurred. This decline is characterized by a loss in first-phase insulin secretion [23]. In the VA Genetic Epidemiology Study, individuals with impaired fasting glucose and impaired glucose tolerance averaged a 70% decline in pancreatic insulin secretion compared with individuals with normal glucose tolerance, after correcting for variable degrees of insulin sensitivity [24]. Since the primary goal of type 2 diabetes prevention is preserving β-cell function [25], the glucose criterion of MetS is inadequate for the early detection of diabetes risk.

Elevated fasting triglyceride level, another MetS criterion, provides an alternative marker of early insulin resistance. However, the cutoff value may not be properly calibrated. In a study of 178 normoglycemic adults from New Zealand, 42% of whom had insulin resistance, the optimal triglyceride cutoff value was 1.5 mM (133 mg/dL), rather than 1.7 mM (150 mg/dL) as specified in the MetS criteria. The 1.5 mM cutoff was rigorously calibrated against the euglycemic clamp, a direct measure of insulin sensitivity [26]. Waist circumference is another MetS criterion related to insulin resistance and obesity. However, as acknowledged by the Cardiometabolic Health Alliance, waist circumference is an imperfect gauge of the ectopic lipid deposition and visceral adiposity associated with type 2 diabetes risk [5].

With respect to atherosclerosis, early plaque formation is driven by inflammation and cholesterol deposition in the arterial wall [16, 27]. Yet, the MetS clinical criteria do not include markers of inflammation and elevated cholesterol [4]. The prothrombotic state, characterized by increased fibrinogen, platelet and PAI-1 levels, also contribute to plaque progression and the triggering of cardiovascular events [28–30]. The MetS criteria do not include these measures either. One possible solution is to expand the harmonized definition of MetS into a larger, more comprehensive biomarker panel. However, with added measurements comes complexity and cost, which can render the use of biomarker panels impractical for population screening and front-line clinical monitoring. There is an unmet need for simpler, more effective approaches.

Here we present an atypical new biomarker for MetS that does not rely on direct measures of glucose, triglycerides, waist circumference, cholesterol, inflammatory markers or biomarker panels. Rather, it is based on the motional properties of water—by far, the most abundant molecule in the blood. Changes in the rotational and translational diffusion of water in plasma or serum can be monitored by T_2_, the transverse relaxation time constant. It can be measured using a simple benchtop implementation of nuclear magnetic resonance relaxometry [31].

This approach exploits the unique properties of water as a metabolic surveillance system, as water molecules form hydrogen bonds with virtually every protein and lipoprotein in plasma or serum. Each protein affects water T_2_ in a specific manner, depending its molecular weight, shape, water-binding properties and concentration [31]. A shift in the concentrations of a *cassette* of proteins and lipoproteins, as occurs in MetS, alters water mobility and reduces water T_2_ values. Thus, water T_2_ simultaneously monitors the net response to changes in many blood proteins, providing a global view of an individual’s metabolic state with just one measurement. The measurement can be made in a few minutes using a small volume of unmodified human plasma or serum, and requires no chemical reagents or reactions. Pending further testing and validation, water T_2_ offers a surprisingly powerful, yet practical new tool for detecting metabolic syndrome and monitoring cardiometabolic health.

## Methods

### Study design

This was a biomarker discovery study with an observational, cross-sectional design. Initially, it was designed to test the hypothesis that serum water T_2_ was associated with markers of insulin, glucose and lipid metabolism. Phase 1 was designed to collect data on 29 non-diabetic human subjects. The target number of subjects was derived from a power calculation with α = 0.05, β = 0.2 (statistical power of 0.8) and a correlation coefficient of 0.5 [32, 33]. The actual number of subjects analyzed in Phase 1 was 28.

Analysis of the Phase 1 data led to the observation that, in addition to insulin-, glucose- and lipid-related markers, water T_2_ appeared to be correlated with inflammatory markers. So Phase 2 of the study added an expanded set of inflammatory and acute phase markers. The target number of 38 subjects for Phase 2 was derived from a power calculation with α = 0.05, β = 0.1 (statistical power of 0.9) and a correlation coefficient of 0.5 [32, 33]. The actual number of subjects analyzed in Phase 2 was 44.

Therefore, the total number of subjects analyzed and reported here was 72: 28 in Phase 1 and 44 in Phase 2. Many biomarkers were collected in both Phase 1 and 2, while some were collected only in Phase 2.

### Subject recruitment

Human subject volunteers were recruited with prior written informed consent into two protocols approved by the Institutional Review Board of the University of North Texas Health Science Center in Fort Worth (UNTHSC). One protocol recruited adult subjects from the student and staff population of UNTHSC, including spouses, friends and associates. The second protocol recruited Fort Worth community members enrolled in the Health & Aging Brain Study at UNTHSC [34]. Exclusion criteria for the current study included diabetes (HbA_1C_ ≥ 6.5, fasting plasma glucose ≥ 125 mg/dL or prior history/diagnosis), active acute or chronic illness (C-reactive protein > 10 or history/diagnosis), history of bleeding disorders or difficulty donating blood, confirmed or suspected pregnancy from medical history, or not fasting for 12 h. Inclusion criteria were ages 18 and up. A total of 87 subjects were enrolled in the study, with 72 of the 87 subjects qualified according to the inclusion and exclusion criteria.

All subjects completed a comprehensive medical history form and a follow-up interview prior to the day of blood draw. On the morning of the blood draw, anthropometric measurements (height, weight, waist circumference, blood pressure and heart rate) were taken by the study nurse, and urine samples were screened for microalbuminuria using Chemstrip Micral (Roche Diagnostics).

### Blood collection

Fasting blood samples were drawn at 7 a.m. by the study nurse following a standard order-of-draw protocol. For plasma preparation, blood was drawn into BD Vacutainer lavender-top tubes containing K_2_EDTA as the anticoagulant. For serum used for NMR and viscosity measurements, blood was drawn into plain glass red-top tubes lacking any gel separator or clot activators (BD models 366,441 and 366,430) to avoid potential interference in the NMR and viscosity measurements. Every effort was made to collect enough blood to perform all planned measurements. However, there were instances where the amount of blood collected from a given subject was not sufficient or samples were rejected by the testing lab due to hemolysis or other reasons. That variability, along with a few laboratory errors (instrument malfunction, data not collected or accidentally overwritten), accounted for the test-to-test differences in sample size (n) for the measurements listed in the tables. No attempts were made to interpolate or fill in missing data.

### Blood sample processing, analysis and bio-banking

The plasma and serum samples were processed immediately after each blood draw. The serum samples were allowed to clot for 30 min, while plasma samples were being centrifuged. The first spin was at 3380 rpm (1590×*g*) for 10 min at room temperature to pellet and remove blood cells, followed by a second spin of the supernatant at 3800 rpm (2361×*g*) for 15 min to remove residual cells or debris. The presence of residual platelets was ruled out by dynamic light scattering analysis of each twice-centrifuged sample using a Wyatt Mobius instrument. The water T_2_ measurements were performed in triplicate on a sample of fresh plasma followed immediately by three repeats on fresh serum such that all water T_2_ measurements were completed within 2 h after the blood draw. Likewise, viscosity was measured on fresh serum and plasma samples within a few hours of the blood draw using a VISCOLab3000 instrument [35]. Aliquots of fresh serum were sent on ice to Atherotech, Inc. for Vertical Autoprofile (VAP) advanced lipoprotein testing and to determine LDL-P, hs-CRP, GGT, homocysteine, and Lp(a). Other aliquots of fresh plasma and serum were temporarily stored at 4°C prior to being sent the same day to Quest or Labcorp for diagnostic testing. Plasma aliquots for amino acid analysis, glucagon, fibrinogen, free fatty acids and proinsulin were frozen immediately after preparation and stored at − 80 °C prior to shipment to Quest. Other individual aliquots of plasma and serum were frozen at − 80 °C for subsequent in-house analysis using the following assay kits: apolipoprotein E concentration (Abcam, Ab108813), ORAC antioxidant capacity (Cell Biolabs, STA-345), protein carbonyl content (Cell Biolabs, STA-307), HNE (Cell Biolabs, STA-838), phospholipids (Wako Diagnostics, Assay Kit C), α_2_-macroglobulin (Abcam, ab108888), PAI-1 (Abcam, ab184863), neutrophil elastase (Abcam ab119553), soluble fibronectin (Abcam, ab181419), l-lactate (Abcam ab65331), endotoxin (Thermo Scientific, PI88282), and staphylococcus enterotoxin (Creative Diagnostics, DEIA-CL032).

Cytokines were assayed using the V-PLEX platform from Meso Scale Discovery, with a customized human cytokine plate for IL-6, IL-1β, TNF-α and IL-10 (MSD, K151AOH-1). Adiponectin and Factor VII were assayed using MSD Plate K151BXC-1, and sICAM-1, using MSD Kit K151SUD-1. All tests using − 80 °C frozen specimens were performed on samples that underwent only one freeze–thaw cycle.

### Benchtop nuclear magnetic resonance relaxometry

Measurements of T_2_, the transverse relaxation time constant, were performed at 37 °C using a Bruker Minispec mq20 benchtop time-domain NMR instrument equipped with a 10 mm variable temperature probe (Model H20-10-25-AVGX). The 10 mm-diameter sample tube included a 3 mm coaxial insert (Norell NI10CCI-B), and the insert was filled to a sample height of 1 cm, corresponding to a sample volume of ~ 50 μL.

The modified Carr–Purcell–Meiboom–Gill (CPMG) pulse sequence we employed for T_2_ measurements is illustrated in Figure 1 of Ref. [31]. In our experience, a critical factor in obtaining high quality NMR relaxometry data with aqueous samples is to avoid radiation damping, particularly when higher magnetic fields and/or larger sample volumes are used (e.g., 10 mm tubes *without* a coaxial insert). The magnitude of the radiation damping depends on the particular instrument and probe design. We determined that the sample size in the coaxial insert was sufficiently small in this instrument to avoid radiation damping. Thus, it was not necessary to use the optional composite 180° pulse and Δ delay shown in the pulse sequence. Other experimental aspects pertinent to NMR data collection and analysis are detailed in Ref. [31].

### Statistical analysis

The bivariate correlations, multiple and logistical regression analyses, categorical means comparisons, receiver operator characteristic curves and principal components analysis with variable clustering were performed using JMP Pro version 13.1 (SAS, Inc.) and GraphPad Prism v. 6.05 (GraphPad Software, Inc.). The guiding principles for the statistical analyses were derived largely from the books by Motulsky and Huber [36, 37]. Regression residuals were analyzed in GraphPad Prism using the strategy outlined by Klingenberger [38].

The bivariate correlation coefficients were calculated using three complementary methods: Pearson product-moment r, Spearman ρ, and the Huber M-value [36, 37]. These three estimators involve different assumptions about the data, and thus, have different strengths and weaknesses. Included among the assumptions for the Pearson r analysis is that both measures are sampled from a Gaussian distribution [36]. Analysis of the variables in this study revealed that more than half were not Gaussian distributed, as assessed using the D’Agostino–Pearson omnibus normality test implemented in GraphPad. However, a natural log transformation corrected the problem in nearly all cases (see Table 3, footnote c).

Another key assumption of the Pearson and Spearman correlations is that there are no outliers. Pearson is especially sensitive to outliers [36, 37, 39], which can lead to an over- or under-estimation of correlation coefficients. The Huber M-value has the distinct advantage of being robust to outliers [37]. Therefore, we chose to not eliminate any outliers, with the caveat that the Pearson and Spearman coefficients would be interpreted together with the Huber M-values and with careful inspection of the scatter plots.

In most cases, all three correlation coefficients had comparable values. Specific cases where outliers appeared to cause a significant under-estimation of the Pearson or Spearman coefficients included HbA1c, serum % globulins, VLDL-C, Rem-C, platelet count, lymphocyte count, RDW, complement C4c, and anion gap, uncorrected. Cases where outliers may have caused an overestimation of Pearson and Spearman values were asparagine, PAI-1, and haptoglobin. For these reasons, the Huber M-value was taken as the single-best estimate of the correlation, especially in cases where outliers had an influence.

The use of all three methods was particularly useful when correlations were ambiguous, i.e., when one method yielded a statistically significant result, while others did not. One notable example was body-mass-index or BMI, where a weak but statistically significant Pearson correlation was observed with both plasma and serum water T_2_. However, the Spearman and Huber M-value coefficients were weaker and not statistically significant. Inspection of the scatter plots revealed that the Pearson analysis was heavily influenced by a single outlier point that fell well outside of the Huber 95% confidence ellipse. This point corresponded to the subject in this study with the highest BMI and one of the lowest water T_2_ values. However, this study contained mostly non-obese individuals. Therefore, a proper assessment of the possible correlation between BMI and water T_2_ values *in the context of obesity* will require the study of a larger number of obese subjects. In the current study population, water T_2_ did not correlate with BMI.

Multiple linear regression models were built from Gaussian-distributed variables using the stepwise analysis feature in JMP v. 13.1. Potential predictor variables were chosen from the output of the bivariate analyses (Table 3, Additional file 1: Tables S1, S2), and from the principal components analyses with variable clustering, i.e., the most representative variable in each cluster. The stepwise analysis provided starting points for the exploration of different models and the reduction of possibilities. Acceptable models satisfied all three of the following criteria: (1) the p values for all predictor variables in the model were significant at α = 0.05, (2) the model avoided overfitting, as assessed using k-fold cross validation with k = 10, and (3) the adjusted R^2^ was maximized, within the constraints of criteria (1) and (2). The highest number of predictor variables in our models was five, including the y-intercept. This number is consistent with the rule of thumb that models should contain no more than 1 predictor variable for every 8–10 observables (subjects).

For the comparison of two means, unpaired two-tailed t-tests were used to assess significance, assuming equal variances and that the data were sampled from a Gaussian distribution. Five tests to confirm equal variances (O’Brien, Brown–Forsythe, Levene, Bartlett and 2-sided F-test) were performed as implemented in JMP 13.1. In one case specified in Table 4, equal variances could not be confirmed, so significance was assessed using the Welch test instead of the *t* test.

Receiver operator characteristic (ROC) curves were generated and evaluated using JMP 13.1. For determining the sensitivity, specificity and cutoff values for plasma and serum water T_2_, the McAuley Index value of ≤ 6.07 was chosen as the categorical reference standard for early insulin resistance [26]. The equation for the McAuley Index is provided in the abstract of Ref. [26], and the input values of fasting insulin and triglyceride are provided in Table 3 (“Method A”) of Ref. [26]. The optimal ROC cutoff points for plasma and serum water T_2_ were those that fell closest to the [0,1] coordinate, i.e., those which intersected with or closely approached the gray 45° tangent line shown for each ROC curve.

## Results

### Characteristics of the study population

The clinical characteristics of the human study population are presented in Table 1. Overall, this was a fairly diverse group of asymptomatic, non-diabetic adult volunteers spanning a wide age range. The gender distribution was approximately equal. The 72 subjects included 35 white, 23 Asian, 10 Hispanic and 4 African American individuals. The inclusion and exclusion criteria are specified in “Methods” section. As shown in Table 1, the mean values for the diagnostic markers fell near the middle of their normal reference ranges. With respect to glucose markers, 47 of the 72 subjects were normoglycemic by American Diabetes Association criteria [40], with both fasting glucose < 100 mg/dL and HbA_1c_ < 5.7%. The remaining 25 subjects had glucose and/or HbA_1c_ values consistent with prediabetes.

**Table 1:** Characteristics of the study population, n = 72

Parameter	Mean ± S.D.^a^	Range^a^	Reference values^b^
Age	39.5 ± 15.3	23–80	n/a
Gender	n/a	34 female, 38 male	n/a
Body-mass index (kg/m^2^)	26.1 ± 4.9	18.2–45.1	< 25 normal weight, 25–30 overweight, > 30 obese
Plasma T_2_ (ms)	764.4 ± 58.7	631–887	≥ 745.0^c^
Serum T_2_ (ms)	818.4 ± 56.7	692–927	≥ 811.8^c^
Glucose (mg/dL)	90.9 ± 7.7	71–115	< 100 non-diabetic 100–124 (pre-diabetic)
HbA_1c_ (%)	5.5 ± 0.3	4.7–6.2	< 5.7 (non-diabetic) 5.7–6.4 (pre-diabetic)
Insulin C-peptide (ng/mL)	2.0 ± 0.9	0.7–5.1	0.8–3.9 (> 2.85, IR^d^)
Insulin (μU/mL	9.1 ± 6.0	2.2–40.1	2.0–19.6 (> 12.2, IR^d^)
Total serum protein (g/dL)	7.1 ± 0.4	6.2–8.0	6.1–8.1
Serum albumin (g/dL)	4.5 ± 0.3	3.6–5.1	3.6–5.1
Serum globulins (g/dL)	2.7 ± 0.4	1.8–3.3	1.9–3.7
Triglycerides (mg/dL)	117.6 ± 60.0	42–321	< 150
Total cholesterol (mg/dL)	187.0 ± 41.0	97–291	< 200
HDL-C (mg/dL)	53.3 ± 12.7	31–85	≥ 40 (male); ≥ 50 (female)
LDL-C (mg/dL)	111.1 ± 34.7	42–191	< 130
WBC count (×10^3^/μL)	6.5 ± 1.6	3.9–11.2	3.8–10.8
Neutrophil count (×10^3^/μL)	3.6 ± 1.2	1.8–7.3	1.5–7.8
*hs*-CRP (mg/L)	2.3 ± 2.3	0.1–9.6	< 3.0 (low-to-average CV risk) 3.0–10.0 (high CV risk) > 10.0 (infection/illness)
Sodium (mmol/L)	139.0 ± 2.7	131–146	135–146
Potassium (mmol/L)	4.2 ± 0.3	3.5–4.8	3.5–5.3
Total CO_2_, serum (mmol/L)	24.0 ± 2.3	16–29	19–30

^a^All blood samples were collected in the early morning after a 12-h overnight fast

^b^Reference values from Quest Diagnostics and Atherotech, except where noted

^c^Cutoff for normoglycemic population established in this study

^d^Insulin cutoff from McAuley et al. [26]; insulin C-peptide cutoff established by linear regression with inulin

### Bivariate correlations between water T_2_ and blood biomarkers

The plasma and serum water T_2_ values were Gaussian distributed and exhibited high coefficients of variation (7–8%) across the study cohort (Table 1). This high variance did not result from imprecision in the NMR relaxometry measurements, as the coefficient of variation for multiple repeats on a single subject averaged < 1%. Rather, the high variance was caused by subject-to-subject biological variation reflecting the range of metabolic health among the subjects.

To identify the specific factors governing this variation, up to 130 diagnostic blood biomarkers were measured for each subject and correlated with plasma and serum water T_2_ values. As detailed in Methods, the study was conducted in two phases, with many biomarkers measured in both phases (n = 72), and some additional biomarkers measured only in Phase 2 (n = 44). While a number of markers showed significant correlations with plasma water T_2_ (Table 2, middle column), many others did not (Table 2, right column). Among those showing no correlation were albumin and sodium, markers of a subject’s hydration status. In addition, markers related to paramagnetic ions or their binding proteins (transferrin, total iron, total iron binding capacity, percent iron saturation, ferritin and ceruloplasmin) showed no correlation with plasma or serum T_2_, in spite of the inherent sensitivity of T_2_ to changes in paramagnetic ions. Thus, in this study population, variation in water T_2_ was not associated with variation in the subject’s hydration state or iron/copper status.

**Table 2:** Biomarkers measured in this study; TD-NMR markers: plasma water T_2_, serum water T_2_

Category	Correlation with plasma water T_2_^a^	No correlation with plasma water T_2_^a^
Insulin resistance, diabetes and anthropometric markers	Fasting insulin, insulin C-peptide, proinsulin, HbA_1c_, glucose, HOMA-IR, QUICKI, FIRI, G/I ratio, McAuley Index	Body-mass index, waist circumference, age, resting heart rate
Protein, viscosity liver function and amino acid markers	Total serum and plasma protein, serum and plasma globulins, % globulins, viscosity, ALT, GGT, Ser, Asn, Gln, Thr, Tyr	Serum and plasma albumin, IgG, IgM, IgA, AST, homocysteine, and 30 other amino acid and amino acid metabolites
Lipid and lipoprotein markers	Total cholesterol, non-HDL-C, LDL-C, LDL-P, VLDL-C, IDL-C, remnant-C, apoB, phospholipids, TG, TG/HDL ratio	Lp(a), HDL-C, HDL_2_, HDL_3_, apoAI, apoE, omega-3 index, DHA, AA, EPA, LpPLA_2_, serum turbidity (O.D._550 nm_), free fatty acids
Inflammation, acute phase proteins, blood cell and oxidative stress markers	hs-CRP, WBC, neutrophils, lymphocytes, platelets, RDW, MCH, sedimentation rate, fibrinogen, complement C3c, C4c, plasminogen activator inhibitor-1 (PAI-1), α_1_-acid glycoprotein, interleukin-6	RBC, HCT, Hb, MCHC, MCV, mean PLT volume, HNE, ORAC total antioxidant capacity, monocytes, eosinophils, basophils, haptoglobin, fibronectin, transferrin, ceruloplasmin, total iron, TIBC, % iron sat., ferritin, sICAM1, adiponectin, factor VII, uric acid, neutrophil elastase, endotoxin, staph. enterotoxin, α_2_-macroglobulin, α_1_-antitrypsin, IL-10, TNFα, IL-1β
Electrolyte markers	Lactate, Cl^−^, Cl^−^ + CO_2_ (bicarbonate), anion gap, anion gap corrected for albumin	Sodium, potassium, calcium
Kidney and thyroid markers		BUN, creatinine, eGFR, thyroid stimulating hormone (TSH), free T4

^a^A correlation is defined as p < 0.05 for all three correlation coefficients: Pearson, Spearman, and Huber M-value. For biomarkers in the middle column, the individual coefficients and statistics are provided in Table 3 and Additional file 1: Table S1. A list of abbreviations is provided below

**Fig. 1:**
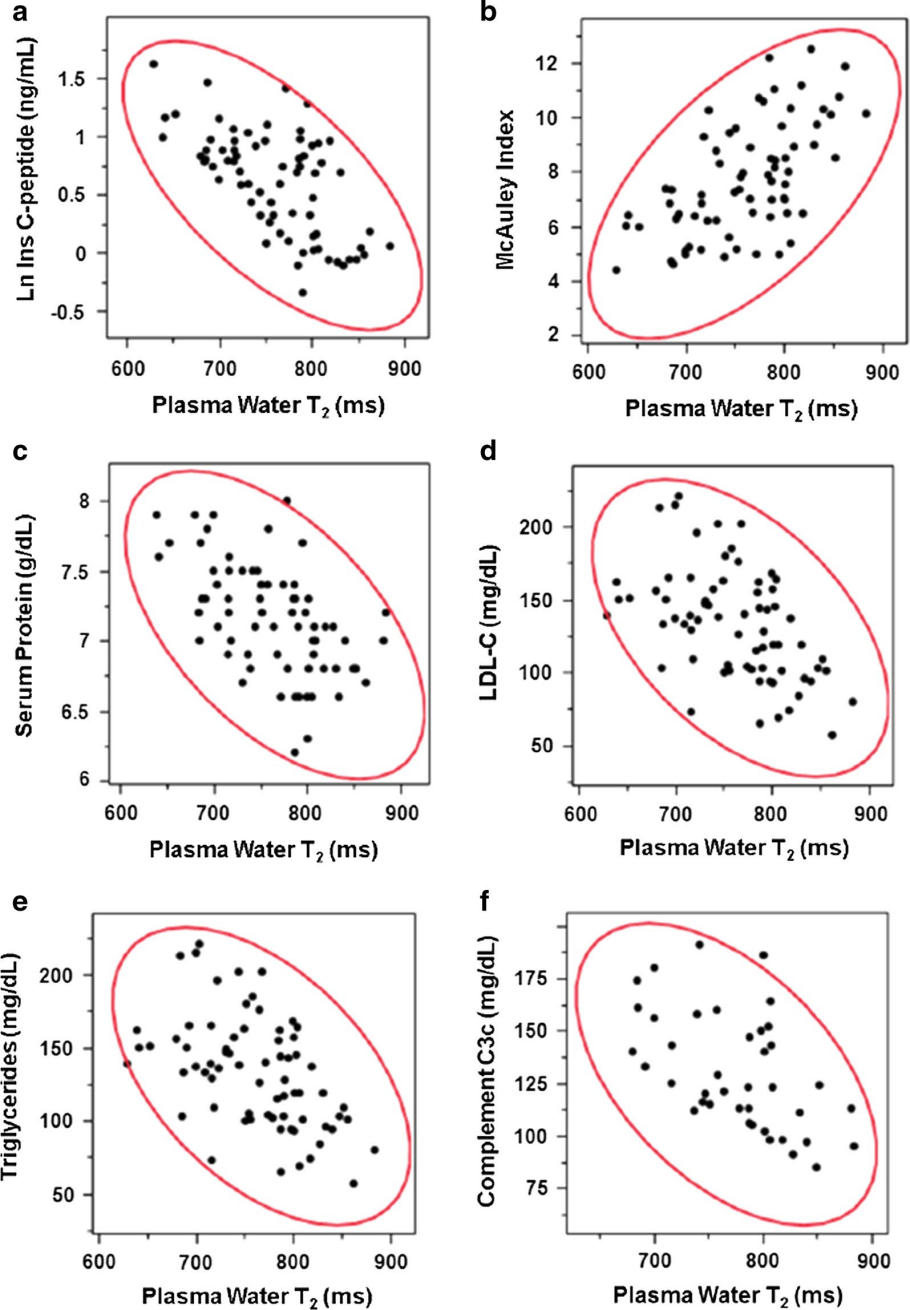
Scatterplots for the bivariate correlations between plasma water T_2_ values and 6 diagnostic markers:** a** natural log of insulin C-peptide;** b** McAuley Index;** c** total serum protein;** d** low-density lipoprotein cholesterol;** e** triglycerides;** f** complement C3c. Each filled black circle represents a data point for an individual human subject. The red ellipse represents the Huber bivariate normal density at the 95% confidence level

Bivariate scatterplots for the correlation between plasma water T_2_ and insulin C-peptide, the McAuley Index, total serum protein concentration, LDL-cholesterol, triglycerides and complement C3 (measured clinically as its stable conversion product C3c) are displayed in Fig. 1. The bivariate normal density ellipse, indicated in red, provides a visual indicator of the Huber correlation at the 95% confidence level. The Huber M-value has the advantage of being robust to outliers, as compared with the Pearson r and the Spearman ρ values [37, 41]. This enabled us to analyze the correlations without excluding any real or perceived outliers, as detailed in “Methods” section.

Table 3 lists the statistically significant bivariate Huber correlation coefficients for biomarkers that were unambiguously correlated with both plasma and serum water T_2_. Strong correlations (0.5–0.7) were observed between water T_2_ and fasting insulin C-peptide, insulin, and proinsulin, as well as indices derived from insulin plus glucose, or insulin plus triglycerides (Table 3, first section). Note that the correlation with fasting glucose, while statistically significant, is considerably weaker (~ 0.3). Also, strong correlations were observed with protein markers, specifically plasma and serum globulins, total serum and plasma proteins, plasma and serum viscosity, as well as alanine aminotransferase, a marker of liver function (Table 3, second section). However, no significant correlations were observed with plasma and serum albumin, which account for ~ 60% of the total plasma and serum protein mass.

**Table 3:** Bivariate Huber correlation coefficients for water T_2_

Biomarker^a,b,c^	n	Plasma water T_2_	n	Serum water T_2_
Insulin and glucose markers				
McAuley Index^d^	70	+ 0.64****	69	+ 0.70****
Insulin C-peptide	70	− 0.65****	69	− 0.45****
HOMA-IR^d^ (insulin c-peptide)	70	− 0.64****	69	− 0.46****
Insulin	70	− 0.57****	69	− 0.60****
HOMA-IR^d^ (insulin)	70	− 0.56****	69	− 0.58****
QUICKI^d^	70	+ 0.60****	69	+ 0.57****
FIRI^d^	70	− 0.58****	69	− 0.59****
Glucose/insulin ratio^d^	70	+ 0.55****	69	+0.58****
Glucose	70	− 0.28*	69	− 0.28*
HbA1c	69	− 0.54****	69	− 0.43***
Proinsulin	42	− 0.53***	43	− 0.60***
Protein, viscosity and liver function markers				
Total protein, serum	69	− 0.56****	68	− 0.79****
Serum globulins	69	− 0.53****	68	− 0.65****
Serum viscosity	65	− 0.30*	67	− 0.45***
Total protein, plasma	41	− 0.55***	42	− 0.72****
Plasma globulins	41	− 0.66****	42	− 0.69****
Plasma viscosity	51	− 0.47***	52	− 0.56****
Alanine aminotransferase (ALT)	52	− 0.37**	50	− 0.35**
Lipid and lipoprotein markers				
Apolipoprotein B (apoB)	70	− 0.55****	69	− 0.52****
Non-high-density lipoprotein cholesterol	70	− 0.52****	69	− 0.52****
Low-density lipoprotein cholesterol (LDL-C)	70	− 0.50****	69	− 0.53****
LDL/HDL ratio	70	− 0.54****	69	− 0.58****
Total cholesterol	70	− 0.50****	69	− 0.51****
LDL particle number (LDL-P)	70	− 0.52****	68	− 0.54****
Triglycerides (TG)	70	− 0.54****	69	− 0.54****
TG/HDL ratio	70	− 0.46****	69	− 0.49****
Phospholipids	65	− 0.44***	66	− 0.41**
Very low-density lipoprotein-chol. (VLDL-C)	63	− 0.44***	62	− 0.49****
Intermediate-density lipoprotein-chol. (IDL-C)	63	− 0.39**	62	− 0.50****
Remnant-cholesterol (Rem-C)^e^	63	− 0.44***	62	− 0.53****
Apo B/Apo A–I ratio	63	− 0.53****	62	− 0.56****
Inflammation and blood cell markers				
White blood cell count (WBC)	69	− 0.58****	68	− 0.47****
Neutrophil count	69	− 0.41***	68	− 0.37**
Lymphocyte count	69	− 0.40***	68	− 0.36**
C-reactive protein (CRP)	69	− 0.51****	68	− 0.31**
Serum % globulins	69	− 0.46****	68	− 0.50****
Plasma % globulins	41	− 0.56***	42	− 0.45**
Fibrinogen	43	− 0.65****	44	− 0.40**
Complement C3c	40	− 0.52***	41	− 0.44**
Complement C4c	40	− 0.59****	41	− 0.43**
Electrolyte markers				
Lactate	41	− 0.53***	42	− 0.49***
Anion Gap, uncorrected	69	− 0.55****	68	− 0.43***
Anion Gap, corrected for [albumin]	68	− 0.44***	67	− 0.39***
Cl^−^ + CO_2_ (HCO_3_^−^)	69	+ 0.36**	68	+ 0.30*

* p < 0.05; ** p < 0.01; *** p < 0.001; **** p < 0.0001

^a^All blood samples were collected in the early morning following a 12-h overnight fast

^b^This table includes only those biomarkers that were unambiguously correlated with both plasma and serum water T_2_. Results for markers that correlated with plasma or serum water T_2_, but not both, are discussed in the text and provided in Additional file 1: Tables S1 and S2. Ambiguous correlations are discussed in “Methods” section

^c^Many of the variables were natural-log transformed in order to meet the normality condition, an assumption inherent to the Pearson correlation. The correlation coefficients reported here and in Additional file 1: Tables S1 and S2 are for the ln-transformed variables, except the McAuley Index and QUICKI, as these indices are inherently ln-transformed. Other variables that were normally distributed and analyzed without ln transformation were plasma and serum T_2_, total serum and plasma protein, serum and plasma globulins and % globulins, serum and plasma viscosity, HbA1c, LDL-C, LDL-P, total C, apolipoprotein B, lymphocyte and platelet counts, lactate, and complement C3c

^d^As defined elsewhere: McAuley Index [26], HOMA-IR [66, 67], FIRI [68], QUICKI [69], and G/I ratio [70]

^e^Remnant cholesterol is defined as intermediate-density lipoprotein (IDL) plus VLDL_3_, as determined using the vertical autoprofile method [71]

Correlation coefficients of ~ 0.4 to 0.6 were observed between water T_2_ and markers of cholesterol- and triglyceride-rich lipoproteins (Table 3, third section). Moreover, correlations were observed with markers of inflammation and coagulation (Table 3, fourth section), especially white blood cell count, fibrinogen, complement C3c and C4c, and C-reactive protein. Finally, statistically significant correlations were observed with electrolyte markers, namely lactate, total measured anions (Cl^−^ + HCO_3_^−^) and the anion gap (Table 3, last section). Electrolyte abnormalities have been associated with insulin resistance, inflammation and high blood pressure in the National Health and Nutritional Examination Survey [42–44].

The association between plasma and serum water T_2_ was very strong, with a correlation coefficient of 0.8. However, some biomarkers correlated with plasma, but not serum, water T_2_ or vice versa. Those that correlated only with plasma water T_2_ include platelet and monocyte counts, red cell distribution width, mean corpuscular volume, mean corpuscular hemoglobin, serine, asparagine, glutamine, threonine, β-alanine, chloride, gamma glutamyl transferase, erythrocyte sedimentation rate, plasminogen activator inhibitor-1, α_1_-acid glycoprotein and interleukin-6. Those that correlated only with serum water T_2_ included immunoglobulin G, lipoprotein-associated phospholipase A_2_, red blood cell count, tyrosine and 3-methyl histidine. Full sets of Pearson, Spearman and Huber correlation coefficients are provided in Additional file 1: Tables S1 and S2 for plasma water T_2_ and serum water T_2_, respectively.

The overall pattern of correlations is consistent with key elements of insulin resistance and the metabolic syndrome, namely hyperinsulinemia, dyslipidemia, pro-inflammation, pro-coagulation, and electrolyte imbalances. In all five categories, plasma and serum water T_2_ values were inversely correlated with those metabolic abnormalities.

Of note, water T_2_ measurements did not correlate with body-mass index or waist circumference, at least in this mostly non-obese population. Also, plasma and serum water T_2_ did not correlate with free fatty acid levels or with age. The mean plasma water T_2_ value was lower in women than men, but this difference was not statistically significant (752.1 vs. 775.3, p = 0.104). By contrast, the mean *serum* water T_2_ values were nearly identical in women and men (817.0 vs. 819.8, p = 0.838). The possible gender difference seen with plasma but not serum water T_2_ could be attributed to a higher level of fibrinogen observed for women vs. men, although the gender difference in fibrinogen did not reach statistical significance (289.4 vs. 255.9, p = 0.091).

The inflammatory markers showed weaker correlation coefficients with serum water T_2_ (~ 0.3 to 0.5; Table 3) as compared with plasma water T_2_ (~ 0.4 to 0.7, Table 3). The primary difference between plasma and serum is the absence of fibrinogen or Factor I in serum [45], providing an explanation for the weaker correlations observed for serum water T_2_. Fibrinogen is a key reporter that connects plasma water T_2_ with inflammation and coagulation status. However, fibrinogen is not the only such reporter, as discussed below.

### Principal components analysis with variable clustering

The observed bivariate correlations led us to consider factors that may contribute directly to the variation in plasma and serum water T_2_, as well as those that may be indirectly linked through another variable or a network of variables. Human blood plasma is a complex mixture containing hundreds of different proteins and lipoproteins as well as numerous small molecule metabolites. At first thought, de-convoluting these myriad variables would seem hopelessly complex. However, water mobility, and hence water T_2_, is affected mainly by its binding to macromolecules, as the influence of small molecule metabolites such as glucose is negligible [46]. Moreover, the sixteen most abundant proteins and lipoproteins in plasma (albumin, IgG, transferrin, fibrinogen, IgA, α_2_-macroglobulin, apolipoprotein AI, α_1_-antitrypsin, complement C3, IgM, haptoglobin, apolipoprotein B, α_1_-acid glycoprotein, apolipoprotein E, complement C4, and ceruloplasmin) account for > 99% of the total plasma protein mass. Thus, identifying the primary variables that contribute to water T_2_ is not an intractable problem.

We used three approaches to reduce the complexity of the network and tease apart variables that contribute independently and/or additively to the variation in plasma and serum water T_2_. The first approach utilized a principal components analysis with variable clustering [47]. This algorithm reduced the dimensionality by identifying clusters of variables that are most closely related. The results of one such analysis are presented in Additional file 1: Table S3. The statistical clusters correspond largely to the categories of markers based on physiological considerations. In the example shown in Additional file 1: Table S3, cluster 1 represents insulin and glucose markers; clusters 2–4, protein and viscosity markers; cluster 5, lipid and lipoprotein markers, clusters 6–8, inflammation markers; and cluster 9, electrolyte markers. One benefit of this analysis was to define the “most representative variable” in each category, which served as a starting point for building multiple regression models.

### Multiple regression analysis

The second approach to reducing the complexity of the variable network used multiple regression to control for the effect of confounders and identify variables that have independent contributions to plasma water T_2_. The parameters for four of the best multiple regression models for plasma water T_2_ are provided in Additional file 1: Table S4. Model 1 was derived using variables collected in both phases of the study (72 subjects), while Models 2–4 included at least one variable that was measured only in Phase 2 (44 subjects). These models accounted for approximately two-thirds to three-fourths of the variation in plasma water T_2_. Attempts to add more variables to the models resulted in overfitting, as assessed using k-fold cross validation and described in “Methods” section.

The primary independent contributors to plasma water T_2_ were (1) insulin c-peptide, (2) total serum or plasma protein, plasma globulins, or plasma viscosity, (3) total cholesterol or apolipoprotein B, and (4) white blood cell count or fibrinogen. In general, one biomarker from each of four categories (insulin, proteins, lipids and inflammation) had *independent* contributions to the variation in plasma water T_2_. A key observation was that plasma water T_2_ was correlated with markers of insulin resistance or metabolic syndrome, even after correcting for total serum or plasma protein, serum or plasma globulins, or plasma viscosity. Attempts to correct for BMI and age did not yield statistical significance for those variables. Likewise, variables in the electrolyte category did not display a contribution to plasma water T_2_ independent of the other four categories. However, lactate could be substituted for insulin c-peptide or insulin in models for *serum* water T_2_. Multiple regression models for serum water T_2_ were similar to those for plasma water T_2_. Examples are presented in Additional file 1: Table S5.

### Categorical and logistic regression analyses

The third approach used categorical, rather than continuous, variables to compare means and assess the additivity of contributions to plasma water T_2_. As shown in Table 4, the subjects were categorized as having or not having hyperinsulinemia, dyslipidemia, inflammation or electrolyte abnormalities, using three measures of each condition. In addition, the subjects were categorized as having or not having clinically-defined metabolic syndrome. The differences in mean plasma water T_2_ values were computed for each of the measures and conditions. The differences were greater when two or more conditions were combined. The largest difference in mean plasma water T_2_ values was observed for subjects who had hyperinsulinemia plus dyslipidemia plus inflammation (Table 4, last row). Thus, the lowest plasma water T_2_ values were observed in subjects who had multiple elements of early metabolic syndrome, as the effect on T_2_ has both independent and additive components.

**Table 4:** Mean plasma water T_2_ values for conditions and measures associated with early metabolic syndrome

Conditions and measures^a^	Cutoff value	Mean plasma T_2_ (ms) ± S.E.			
		No	Yes	Δ^b^	Odds ratio^b^
Hyperinsulinemia (H)	Any of 3 below	796.1 ± 7.9	728.7 ± 8.4	67.4****	9.5 (2.9–32.5)****
High fasting insulin^c^	≥ 10.0 μIU/mL	786.6 ± 7.4	721.7 ± 10.2	65.0****	
High insulin C-peptide^c^	≥ 2.3 mg/mL	780.7 ± 8.0	733.1 ± 11.1	47.6***	
Low McAuley Index^d^	≤ 6.07	778.1 ± 7.2	718.1 ± 13.3	60.0***	
Dyslipidemia (D)	Any of 3 below	798.1 ± 8.6	734.3 ± 8.1	63.8****	5.8 (2.3–15.6)****
High non-HDL-C^c^	≥ 149 mg/dL	782.4 ± 7.9	729.8 ± 10.9	52.6***	
Small, dense LDL	Pattern B/AB	778.0 ± 8.1	736.4 ± 11.6	41.6**	
High LDL-P^c^	≥ 1408 nM	779.1 ± 8.0	734.2 ± 11.5	44.9**	
Inflammation (I)^e^	Any of 3 below	808.3 ± 9.4	738.4 ± 7.3	69.9****	10.4 (3.2–35)****
High CRP^c^	≥ 2.5 mg/L	778.4 ± 7.6	725.1 ± 12.9	53.2***	
High WBC count^c^	≥ 6.92 × 10^3^/μL	780.3 ± 8.3	737.3 ± 10.8	43.0**	
High serum globulins^c^	≥ 2.9 g/dL	788.5 ± 7.7	725.8 ± 9.7	62.7****	
Electrolyte abnormal	Any of 3 below	784.8 ± 10.3	747.3 ± 8.8	37.5**	1.6 (1.1–2.3)*
Low (Cl^−^ + CO_2_)^f^	≤ 126 meq/L	776.5 ± 9.0	745.6 ± 10.3	30.8*	
High anion gap^c^	≥ 17.8 meq/L	777.8 ± 7.9	731.6 ± 11.6	46.2**	
High anion gap corr.^g^	≥ 17.7 meq/L	776.8 ± 8.1	742.0 ± 11.3	34.8*	
Metabolic syndrome	Ref. [4]	776.0 ± 7.4	721.6 ± 14.1	54.5**	2.7 (1.2–5.9)**
H + ≥ 1 other condition	See above	796.8 ± 7.6	725.8 ± 8.3	71.1****	15 (4–63)****
H + ≥ 2 other conditions	See above	792.6 ± 6.9	716.5 ± 9.0	76.1****	17 (4–73)****
H + D + I	See above	786.5 ± 6.5	705.0 ± 10.6	81.4****	24 (5–-124)****

* p < 0.05, ** p < 0.01, *** p < 0.001, **** p < 0.0001

^a^This analysis included variables collected in both Phase 1 and Phase 2 of this study (n = 72)

^b^Mean difference (Δ) and odds ratio (95% confidence limits) for the mean difference shown one column to the left

^c^Top tertile of subjects in this study

^d^Obtained using formula in abstract of ref [26], with insulin = 12.2 μIU/mL and triglyceride = 1.5 mM as input

^e^For this row, the Welch test was used in place of the t test, as equal variances could not be confirmed

^f^Total measured anions, where ~ 95% of total CO_2_ is HCO_3_^−^; bottom tertile of subjects in this study

^g^Anion gap corrected for albumin concentration using regression residuals; top tertile of subjects in this study

To further assess the dose–response relationship between the number of metabolic conditions and plasma water T_2_, the subjects were divided into quintiles with respect to T_2_ values, and the average number of conditions for each quintile was calculated on a scale of 0–4. The average number of conditions increased from 0.71 (top quintile) to 1.57, 2.36, 2.79, and 3.57 for the 4th, 3rd, 2nd and lowest quintiles of plasma water T_2_, respectively. The lower the T_2_ value, the greater the number of conditions associated with metabolic syndrome.

To further quantify the association of plasma water T_2_ with these conditions, logistic regression models were constructed with each of the conditions in Table 4 serving as the categorical outcome. Each model was adjusted for potential confounders, specifically BMI, age and gender. For hyperinsulinemia, the unit odds ratio for plasma water T_2_ was 1.034 (95% confidence limits 1.016–1.053, p < 0.0001). Thus, the observed 67.4 ms decrease in mean plasma water T_2_ increased the odds of hyperinsulinemia by a factor of 1.034^67.4^ = 9.5. The corresponding unit odds ratio for serum water T_2_ and hyperinsulinemia was 1.026 (1.011–1.041, p < 0.0001). When dyslipidemia was the categorical outcome, the unit odds ratios were 1.028 (1.013–1.044, p < 0.0001) and 1.030 (1.015–1.047, p < 0.0001) for plasma and serum water T_2_, respectively. This translated into an odds ratio of 5.8 for the decrease in mean plasma water T_2_ associated with dyslipidemia (Table 4).

Even higher unit odds ratios were observed when inflammation was the categorical outcome: 1.034 (1.017–1.052, p < 0.0001) and 1.040 (1.019–1.061, p < 0.0001) for plasma and serum water T_2_, respectively. Thus, the observed 69.9 ms decrease in mean plasma water T_2_ translated into a 10.4-fold increase in the odds of having inflammation (Table 4). Smaller, but statistically significant odds ratios were observed when the categorical outcome was electrolyte abnormalities. Subjects who met the clinical criteria for metabolic syndrome as a whole had lower T_2_ values and higher odds ratios as well (Table 4).

The large decrease in mean water T_2_ values and high odds ratios provide further evidence of the strong association between water T_2_ and elements of the metabolic syndrome.

### Sensitivity and specificity from ROC analysis

**Fig. 2:**
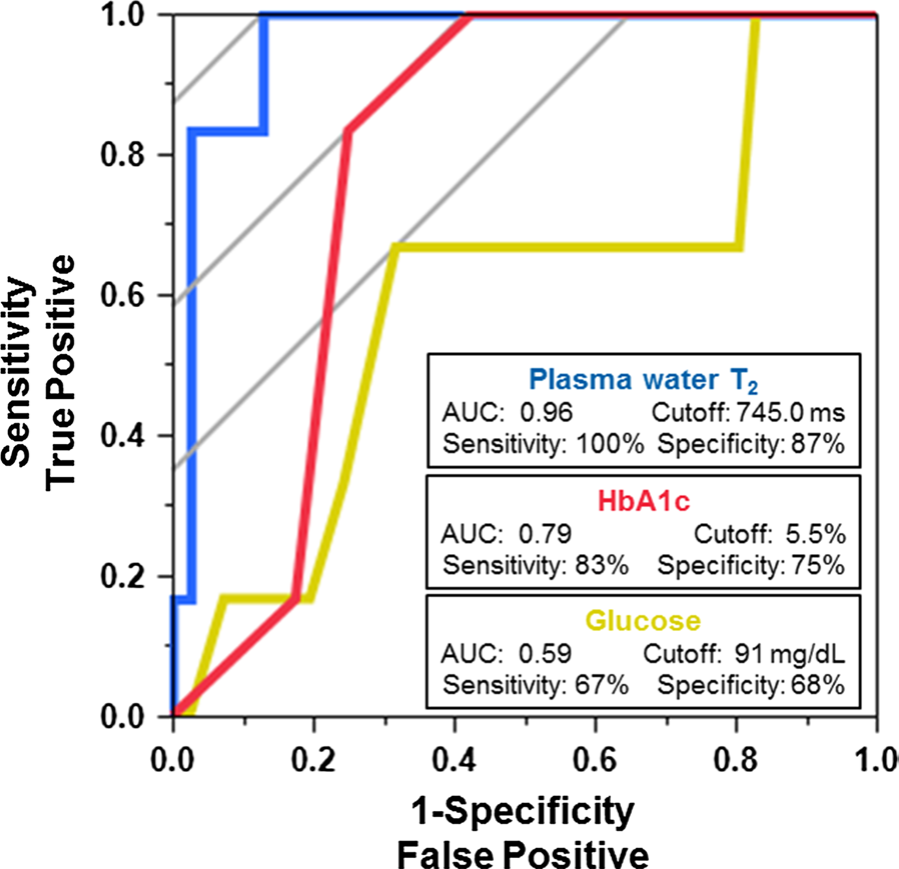
Receiver operator characteristic (ROC) curves to quantify the ability of different diagnostic tests to detect early insulin resistance (metabolic syndrome), as defined by the McAuley Index. Blue (left curve): plasma water T_2_; Red (middle curve): hemoglobin A1c; Yellow (right curve): glucose. An ideal ROC curve follows the left and top axes and intersects with the (0,1 coordinate)

As an initial attempt to assess the sensitivity and specificity of water T_2_ for detecting elements of early metabolic syndrome, we performed a receiver operator characteristic curve (ROC) analysis. Insulin resistance, as defined by the McAuley Index, was used as the reference standard. The McAuley Index combines input from fasting triglycerides and fasting insulin and thus, captures two related elements of MetS. Moreover, the McAuley Index was rigorously calibrated with 178 normoglycemic subjects using a direct measure of insulin sensitivity, obtained from the euglycemic clamp, as the outcome variable [26]. Thus, it was calibrated to detect the earliest stage of insulin resistance. We categorized the 47 normoglycemic subjects in the current study using the McAuley Index cutoff of ≤ 6.07, derived using a fasting insulin of 12.2 μU/mL and a fasting triglyceride of 1.5 mM (133 mg/dL) as input variables [26]. As illustrated by the blue curve in Fig. 2, plasma water T_2_ detects early insulin resistance with a sensitivity of 100% and a specificity of 87% at a cutoff value of ≤ 745.0 ms. The area under the curve (AUC) is 0.96. By contrast, HbA1c and glucose—the tools most widely used for diabetes screening and risk assessment—show lower values of area-under-the-curve (AUC) and lower combinations of sensitivity and specificity (Fig. 2, red and yellow, respectively). To detect early insulin resistance with 100% sensitivity, the HbA_1c_ cutoff would have to be lowered to 5.4, which would lead to poor specificity and a 42% false positive rate.

For plasma water T_2_, the likelihood ratio for a positive test result (LR+) was 7.8. The likelihood ratio for a negative result (LR−) was zero.

Serum water T_2_ yielded ROC curve parameters similar to those of plasma: AUC = 0.94 with a sensitivity of 100% and specificity of 80% at a cutoff value of ≤ 811.8 ms. The LR+ and LR− values were 5.0 and zero, respectively.

As a further exercise, we used the harmonized clinical criteria for metabolic syndrome [4] as the reference standard for ROC analysis, instead of the McAuley Index. For plasma water T_2_, this analysis yielded sensitivity and specificity values of 73% at a cutoff value of 745 ms and an AUC = 0.75. For serum water T_2_, the values were similar: 71% sensitivity and 69% specificity at a cutoff of 804.8 ms and an AUC = 0.71.

### Identification of early metabolic abnormalities using water T_2_

**Fig. 3:**
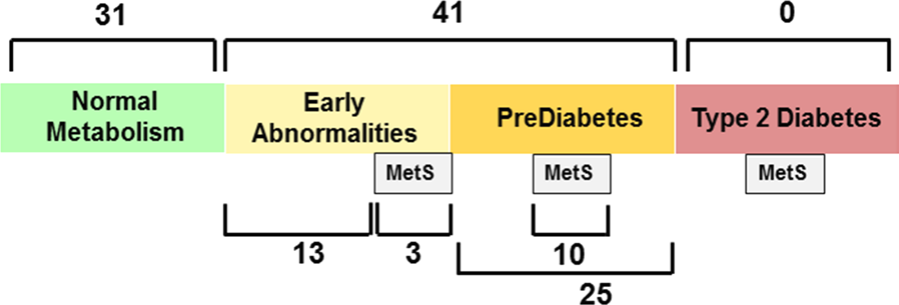
The distribution of the 72 subjects in this study with respect to metabolic abnormalities. Thirty-one subjects had no identifiable metabolic abnormalities (“Normal Metabolism”), while 41 had early abnormalities, prediabetes and/or clinically-defined metabolic syndrome (MetS). Twenty-five of the 41 met the ADA criteria for prediabetes, and 10 of those 25 also met the harmonized clinical criteria for MetS [4]. The remaining 16 subjects with “Early Abnormalities” were identified by low water T_2_ values in the absence of prediabetes. Only three of those 16 met the clinical criteria for MetS. Thus, water T_2_ uniquely identified 13 individuals with abnormalities consistent with early metabolic syndrome

Using the plasma and serum water T_2_ cutoff values of 745.0 and 811.8 ms, along with the current criteria for prediabetes [40] and metabolic syndrome [4], the subjects in this study were classified into metabolic stages, as shown in Fig. 3. Of the total of 72 subjects, 31 had normal metabolism, while the other 41 had early metabolic abnormalities, prediabetes and/or metabolic syndrome defined by clinical criteria. Of the 41, 25 had prediabetes, and 10 of those 25 met the clinical criteria for metabolic syndrome. The remaining 16 did not have prediabetes, but had plasma or serum water T_2_ values below the cutoffs. Of those 16, only three met the clinical criteria for metabolic syndrome. Therefore, plasma and serum water T_2_ uniquely identified 13 subjects (18% of the study population) with metabolic abnormalities that would have gone undetected by the current clinical definitions of prediabetes and metabolic syndrome.

Further examination of the 13 normoglycemic subjects with low plasma or serum water T_2_ values yielded the following observations:

A. Three of the 13 subjects had overt compensatory hyperinsulinemia, with fasting insulin above 12.2 μIU/mL [26] and insulin C-peptide in the top tertile. All three subjects had subclinical inflammation as well.
B. Another 3 of the 13 subjects showed evidence of hyperinsulinemia, but missed the fasting insulin cutoff of 12.2 μIU/mL; two of the three also missed the McAuley Index cutoff < 6.07 [26]. However, they had insulin and insulin c-peptide levels in the top tertiles of the subjects in this study. Two of these three subjects showed evidence of inflammation.
C. Two subjects had low or moderate insulin levels, but insulin c-peptide in the top quartile. The mismatch between insulin vs. insulin c-peptide likely results from rapid hepatic insulin clearance rates. Insulin is cleared by the liver, whereas insulin c-peptide is cleared more slowly by the kidney [48]. Subjects with rapid insulin hepatic clearance may have a limited capacity to sustain insulin levels high enough to compensate for tissue insulin resistance. These subjects may be prone to develop impaired glucose tolerance. In addition to elevated insulin c-peptide, both subjects showed evidence of inflammation.
D. Three subjects showed no evidence of hyperinsulinemia, as monitored by insulin, c-peptide or the McAuley Index, but exhibited high levels of proinsulin, with ratios of proinsulin/insulin c-peptide in the top tertile. This pattern points to a defect in the enzymatic conversion of proinsulin to insulin and insulin c-peptide, which has been observed in non-diabetic and diabetic subjects [49–53]. High proinsulin levels are predictive of incident type 2 diabetes and insulin resistance in diabetes [49, 51, 52]. In addition, these subjects showed signs of dyslipidemia and inflammation.
E. The remaining two subjects had no apparent elevations in insulin, insulin c-peptide or proinsulin. One subject had elevated total cholesterol, LDL-cholesterol and LDL particle number, but not triglycerides or triglyceride-related markers. In addition, this subject had levels of white blood cells and neutrophils in the top tertile, but C-reactive protein and serum globulins were unremarkable. This subject appeared to have a type of dyslipidemia and subclinical inflammation unrelated to insulin resistance. The remaining subject had only three abnormalities besides a low serum T_2_: elevated lipoprotein (a), an increased anion gap and serum globulins in the top quartile.

Thus, the 13 normoglycemic subjects with low water T_2_ had a heterogeneous set of early metabolic abnormalities (hyperinsulinemia, dyslipidemia and/or inflammation) that were undetected by the clinical criteria for MetS. This observation illustrates the limitations of the clinical definition of MetS, and the unique power of water T_2_ to detect this condition.

### Identifying the principal drivers of low water T_2_ in metabolic syndrome

**Fig. 4:**
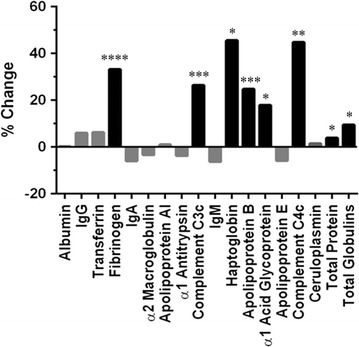
The percent change in mean plasma protein concentration between the low vs. high water T_2_ groups, using the ROC-established cutoffs defined in the text. The 16 most abundant proteins in plasma are listed from left to right in order of decreasing concentration, with total protein and total globulin concentrations listed at the far right. Together, these 16 proteins accounted for > 99% of the total plasma protein mass. *p < 0.05, **p < 0.01, ***p < 0.001, ****p < 0.0001

To assess the role of the 16 most abundant plasma proteins and lipoproteins in metabolic syndrome, the plasma and serum water T_2_ cutoffs of ≤ 745.0 and ≤ 811.8 ms were used to classify the study subjects into those with and without the syndrome. The percent differences in the mean plasma protein concentrations for the two groups of subjects are displayed in Fig. 4. Subjects with metabolic syndrome, here defined by low water T_2_, displayed statistically significant increases in the mean concentrations of fibrinogen, complement C3c, haptoglobin, apolipoprotein B, α_1_-acid glycoprotein and complement C4c, as well as total plasma proteins and globulins (Fig. 4, black bars). By contrast, there were no significant changes in the concentrations of the other 10 proteins: albumin, IgG, transferrin, IgA, α_2_-macroglobulin, apolipoprotein AI (HDL), α_1_-antitrypsin, IgM, apolipoprotein E and ceruloplasmin (Fig. 4, grey bars). Thus, low T_2_ values and metabolic syndrome are characterized by increases in the concentrations of a *specific subset* of acute phase proteins and lipoproteins.

## Discussion

This report describes the serendipitous discovery of a new biomarker for early metabolic syndrome and its initial characterization in human subjects. The results revealed the strong correlations between plasma and serum water T_2_ values and markers of five conditions related to metabolic syndrome: hyperinsulinemia, dyslipidemia, pro-inflammation and pro-coagulation states, and electrolyte imbalances. The correlations were observed in a cohort of otherwise healthy, non-diabetic, mostly non-obese adults. Based on multiple and logistic regression analyses, these conditions had independent and/or additive contributions to the lowering of water T_2_. Water T_2_ values were driven lower by increases in the concentrations of 6 of the 16 most abundant proteins and lipoproteins in human plasma. Five of the six were positive acute phase proteins—markers of innate immunity—and the other was apolipoprotein B, a major protein component of cholesterol- and triglyceride-rich lipoprotein particles.

The five most abundant acute phase proteins associated with lower plasma water T_2_—fibrinogen, complement C3, haptoglobin, α_1_-acid glycoprotein and complement C4—have been the focus of several prospective epidemiological studies. The Framingham Study helped to establish fibrinogen as a risk factor for cardiovascular disease [28], and the Insulin Resistance Atherosclerosis Study demonstrated the relationship between fibrinogen and insulin resistance syndrome (metabolic syndrome) [29]. In addition to its effects on thrombogenesis and platelet aggregation, fibrinogen affects the rheology of blood flow by making the blood more viscous [30], which may explain part of the mechanism by which it lowers plasma blood T_2_ values. In a study of hospital patients, most of whom had severe lung disease (carcinoma, metastases, infectious or inflammatory diseases), an inverse correlation was observed between fibrinogen and plasma T_2_ [54]. The interpretation was that fibrinogen was monitoring the inflammatory status of the patient, rather than the presence or absence of cancer [54]. Those observations are consistent with the current study, even though the current subjects do not have any serious acute or chronic illnesses. The current study highlights the exquisite sensitivity of water T_2_ to detect subtle, subclinical inflammation, even in subjects who are otherwise healthy.

In a prospective study [55], fibrinogen, complement C3, C4 and haptoglobin were associated with insulin resistance and incident type 2 diabetes, but not α_1_-antitrypsin, ceruloplasmin or orosomucoid. This pattern of selective increases in some acute phase proteins, but not others, is generally consistent with the pattern of changes detected here by plasma water T_2_. The current observations highlight the unique capability of water T_2_ to detect changes in a *cassette* of co-regulated acute phase proteins using just one measurement.

Moreover, plasma and serum water T_2_ were inversely correlated with the concentrations of apolipoprotein B and apo B-containing lipoproteins. These abundant nanoparticles constitute the largest molecular assemblies in plasma and serum, ranging from ~ 20 nm diameter for cholesterol-rich LDL, to ~ 60 to 100 nm for triglyceride-rich VLDL. Increased concentrations of apo B-containing lipoprotein particles should cause a profound decrease in water mobility and lowering of water T_2_, as water binds to a larger number of particles. Through the elevation of plasma triglycerides, insulin resistance causes a remodeling of LDL particles, resulting in a larger number of smaller, denser particles [56, 57]. At a given cholesterol level, this increase in particle number provides additional surface area for water molecules to bind and thus, could lower water T_2_ values. In addition, the accumulation of remnant lipoprotein particles in the blood that occurs with insulin resistance could have a similar effect on lowering T_2_.

A curious observation was the *lack* of correlation between water T_2_ and HDL-cholesterol or apolipoprotein A-I, even though HDL-C was inversely correlated with triglyceride levels. A possible explanation is that low HDL-C is not a prominent feature of early metabolic syndrome, as typified by the subjects in this study, but becomes more prominent in later stages of MetS when triglyceride levels tend to be higher, such as overt type 2 diabetes. This distinction is important, as low HDL-C is one of the five clinical criteria for metabolic syndrome.

**Fig. 5:**
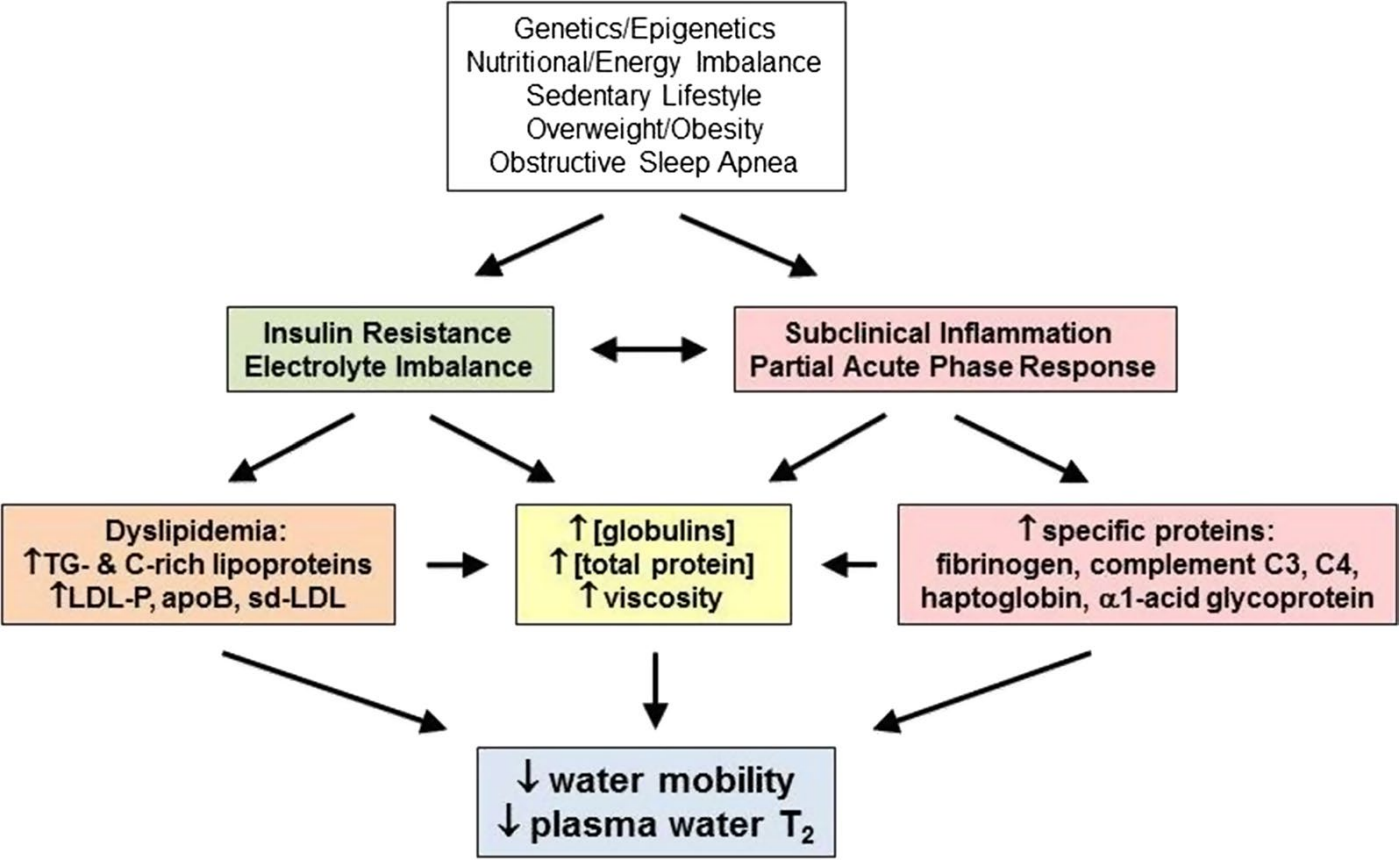
A model for the linkage between genetic and environmental factors, metabolic abnormalities, blood biomarkers, and plasma and serum water T_2_ values

As expected, water T_2_ values were inversely correlated with measures of the bulk properties of plasma and serum, i.e., viscosity and total protein concentration. However, after correcting for bulk factors using multiple regression, water T_2_ remained independently associated with markers of hyperinsulinemia, dyslipidemia and inflammation. Thus, water T_2_ is driven by non-specific changes in bulk factors *as well as* specific changes in individual proteins tied to different aspects of metabolism. A model for how metabolic syndrome reduces water T_2_ is presented in Fig. 5.

Plasma and serum water T_2_ showed a remarkably high sensitivity and specificity for detecting early insulin resistance in subjects with normal fasting glucose and HbA1c levels. The sensitivity was 100%, with no false negatives. The specificity was 86% for plasma and 80% for serum, as water T_2_ detected two individuals with cardio-metabolic abnormalities apparently unrelated to early insulin resistance.

The profile of sensitivity and specificity for water T_2_ makes for a good screening test, which is not the same as a diagnostic test [58]. Screening is beneficial when (i) the disease is serious, (ii) treatment before symptoms is more effective than treatment delayed until after symptoms, and (iii) the prevalence of the detectable pre-clinical phase is high [59]. Type 2 diabetes and atherosclerotic cardiovascular disease meet those criteria. A good screening test is inexpensive, easy to administer, reliable, reproducible, has minimal discomfort and is valid, i.e., sensitive and specific. Water T_2_ meets those criteria. Unlike disease risk surveys, where risk scores are based on broad population averages, water T_2_ provides a *personalized* assessment of metabolic health status. Pending further testing and validation, water T_2_ appears to be a promising screening test for identifying those at risk for prediabetes, type 2 diabetes, atherosclerotic cardiovascular disease, and possibly even Alzheimer’s disease. Approximately one-third of Alzheimer’s cases are preventable, and insulin resistance and diabetes are among the modifiable risk factors [60–64].

The measurement of water T_2_ in human blood is surprisingly simple and practical [31]. The test uses a small volume (~ 50 μL) of unmodified plasma or serum, and requires no reagents, chemical reactions or sample manipulations. The data collection takes ~ 3 min, and the analysis is quick and straightforward. Currently, the measurement is made in a benchtop NMR relaxometry device about the size of a toaster oven, but even smaller devices for this purpose could be designed [31, 65]. Although this study used a research-grade instrument, a clinical instrument designed for the diagnosis of sepsis and blood coagulation in critical care units is commercially available from T2 Biosystems, Inc. (Lexington, Massachusetts, USA). It can perform the measurements described here. It is feasible to perform this test in point-of-care settings like primary care clinics.

### Limitations of the study

This biomarker discovery study provides an initial assessment of the metabolic information content of plasma and serum water T_2_ in non-diabetic human subjects. It needs to be followed up by a series of validation steps: in longitudinal cohorts, in the postprandial state, against direct measures of insulin sensitivity, in larger populations and in response to therapeutic interventions, as explained below.

This cross-sectional study does not provide *direct* evidence for the ability of plasma or serum water T_2_ to predict future risk for type 2 diabetes or cardiovascular disease. However, the individual acute phase proteins and lipoproteins monitored by water T_2_ have been shown, in prior prospective studies, to be associated with incident type 2 diabetes and cardiovascular events. Future work should assess water T_2_ in longitudinal cohorts.

All blood testing was performed on subjects who underwent a 12-h overnight fast. The current results provide little or no direct insight into postprandial metabolism or glucose tolerance following an oral glucose load. Thus, T_2_ will need to be correlated with results from oral glucose tolerance tests.

This study employed indirect measures of insulin resistance based on fasting insulin, insulin c-peptide, proinsulin, triglycerides and indices derived from them, most notably the McAuley Index. An important next step is to validate plasma and serum water T_2_ values against direct measures of tissue insulin resistance.

The correlation of water T_2_ with BMI and waist circumference in the context of obesity was inconclusive, as this study enrolled mainly non-obese subjects. Further work needs to be done to evaluate the association of water T_2_ with BMI and central obesity. Likewise, the relationship of water T_2_ with blood pressure warrants further evaluation.

While the 72 subjects employed in this biomarker discovery study provided sufficient statistical power to identify biologically important correlation coefficients greater than ~ 0.25, a future study with a larger number of subjects will permit a more comprehensive statistical analysis. The main benefit of a larger study would be the generation of multiple and logistical regression models that can accommodate a larger number of predictor variables. Remarkably, we were able to account for three-fourths of the variation in water T_2_ with up to five predictor variables The high sensitivity and large variance in water T_2_ made that possible. However, a study with a larger number of subjects may be able to account for other independent predictors of water T_2_.

## Conclusions

Water T_2_ from benchtop NMR relaxometry offers a new tool for detecting individuals with metabolic syndrome. Its advantages can be summarized in three words: early, global and practical. It detects the earliest abnormalities, capturing a global view of an individual’s metabolic health, with one simple measurement. Water T_2_ should be a central component of personalized strategies to assess metabolic health and prevent type 2 diabetes and atherosclerotic cardiovascular disease.

## Additional file

**Additional file 1: Table S1.** Correlation coefficients for plasma water T_2_-provides a full set of Pearson, Spearman and Huber correlation coefficients for biomarkers that have a statistically significant correlation with plasma water T_2_. **Table S2.** Correlation coefficients for *Serum* water T_2_-provides a full set of Pearson, Spearman and Huber correlation coefficients for biomarkers that have a statistically significant correlation with *serum* water T_2_. **Table S3.** Principal components analysis with variable clustering-provides clusters of related variables and most representative variable in each cluster. **Table S4.** Multiple regression models for plasma water T_2_-provides parameter details for the best multiple regression models for plasma water T_2_. **Table S5.** Multiple regression models for *Serum* water T_2_-provides parameter details for the best multiple regression models for serum water T_2_.

## Abbreviations

AA: arachidonic acid
ALT: alanine aminotransferase
ApoB, apoA–I or apoE: apolipoprotein B, A–I or E concentration
Asn: asparagine
AST: aspartate aminotransferase
AUC: area under the curve in ROC analysis
BMI: body-mass index
BUN: blood urea nitrogen
C3c: complement C3c, a proteolytic fragment of complement C3 measured in diagnostic tests
C4c: complement C4c, a proteolytic fragment of complement C4 measured in diagnostic tests
Cl^−^: chloride anion
CO_2_: total carbon dioxide in serum, ~ 95% of which is in the form of bicarbonate ion or HCO_3_^−^
CPMG: Carr–Purcell–Meiboom–Gill NMR pulse sequence to measure T_2_
DHA: docosahexaenoic acid
EDTA: ethylene-diamine-tetra-acetic-acid
eGFR: estimated glomerular filtration rate
EPA: eicosapentaeneoic acid
FIRI: fasting insulin resistance index
GGT: gamma glutamyl transferase or transpeptidase
G/I ratio: fasting glucose-to-insulin ratio
HABS: Health & Aging Brain Study at the UNT Health Science Center, Fort Worth
HbA_1C_: percent glycated hemoglobin
HCT: hematocrit
HDL_2_ or HDL_3_: high-density lipoprotein, subfractions 2 or 3, cholesterol concentration
HDL-C: high-density lipoprotein cholesterol concentration
HDL-P: high-density lipoprotein particle number concentration
Hb: hemoglobin concentration
HNE: 4-hydroxynonenal, a product of lipid peroxidation
HOMA2-%B: homeostatic model assessment version 2, % beta cell function
HOMA2-%S: homeostatic model assessment version 2, % insulin sensitivity
HOMA2-IR: homeostatic model assessment version 2, insulin resistance index (see https://www.dtu.ox.ac.uk/homacalculator for HOMA2 definitions)
*hs*-CRP: C-reactive protein concentration measured using high-sensitivity assay
IDL-C: intermediate-density lipoprotein cholesterol concentration
IgA, IgG, IgM: immunoglobulins A, G or M
IL-6, -1β, or -10: interleukin-6, -1β, or -10
LDL-C: low density lipoprotein cholesterol concentration
LDL-P: low density lipoprotein particle number concentration
Lp(a): lipoprotein (a) cholesterol concentration
LpPLA_2_: lipoprotein-associated phospholipase A_2_
LR+: likelihood ratio for a positive test
LR−: likelihood ratio for a negative test
M-value: correlation coefficient from Huber M-value estimator
MCH: mean corpuscular hemoglobin (per cell)
MCHC: mean corpuscular hemoglobin concentration (per unit volume of red cells)
MCV: mean corpuscular volume
MDA: malondialdehyde
MetS: metabolic syndrome
NMR: nuclear magnetic resonance
O.D._550_: optical density at 550 nm
ORAC: oxygen radical absorbance capacity, a measure of anti-oxidant capacity
PAI-1: plasminogen activator inhibitor-1
PLT: platelets
QUICKI: quantitative insulin sensitivity check index
RBC: red blood cell count
ROC: receiver operator characteristic
Ser: serine
T_2_: transverse or spin–spin relaxation time constant obtained by NMR relaxometry
T_2a_: regression residuals from a linear fit of plasma or serum water T_2_ vs. serum albumin
T_2c_: regression residuals from a linear fit of plasma or serum water T_2_ vs. serum cholesterol
T_2g_: regression residuals from a linear fit of plasma or serum water T_2_ vs. serum globulins (globulins = total serum protein − serum albumin)
T_2p_: regression residuals from a linear fit of plasma or serum water T_2_ vs. total serum protein
T_2v_: regression residuals from a linear fit of plasma or serum water T_2_ vs. viscosity
TG: triglyceride or triacylglycerol concentration
Thr: threonine
TIBC: total iron binding capacity
Tyr: tyrosine
r: Pearson correlation coefficient, parametric
ρ or r_S_: Spearman correlation coefficient, non-parametric
R^2^: square of the Pearson correlation coefficient
RDW: red cell distribution width
Remnant-C: remnant lipoprotein particle cholesterol concentration
sICAM1: soluble intercellular adhesion molecule 1
T_4_: thyroxine or tetraiodothyronine (thyroid hormone)
TD-NMR: time-domain nuclear magnetic resonance
TG: serum triglyceride concentration
TNFα: tumor necrosis factor alpha
TSH: thyroid stimulating hormone
[UA]: unmeasured anion concentration, in meq/L
[UC]: unmeasured cation concentration, in meq/L
VAP: Vertical AutoProfile test, Atherotech
VLDL-C: very low density lipoprotein cholesterol concentration
WBC: white blood cell count

## References

[1] Cameron AJ, Shaw JE, Zimmet PZ (2004). The metabolic syndrome: prevalence in worldwide populations. Endocrinol Metab Clin North Am.

[2] Aguilar M, Bhuket T, Torres S, Liu B, Wong RJ (2015). Prevalence of the metabolic syndrome in the United States, 2003–2012. JAMA.

[3] Moore JX, Chaudhary N, Akinyemiju T (2017). Metabolic syndrome prevalence by race/ethnicity and sex in the United States, National Health and Nutrition Examination Survey, 1988–2012. Prev Chronic Dis.

[4] Alberti KG, Eckel RH, Grundy SM (2009). Harmonizing the metabolic syndrome: a joint interim statement of the International Diabetes Federation Task Force on Epidemiology and Prevention. Circulation.

[5] Sperling LS, Mechanick JI, Neeland IJ, Herrick CJ, Després J, Ndumele CE, Vijayaraghavan K, Handelsman Y, Puckrein GA, Araneta MRG, Blum QK, Collins KK, Cook S, Dhurandhar NV, Dixon DL, Egan BM, Ferdinand DP, Herman LM, Hessen SE, Jacobson TA, Pate RR, Ratner RE, Brinton EA, Forker AD, Ritzenthaler LL, Grundy SM (2015). The cardiometabolic health alliance. J Am Coll Cardiol.

[6] Grundy SM (2016). Metabolic syndrome update. Trends Cardiovasc Med.

[7] Kahn R, Buse J, Ferrannini E, Stern M (2005). The metabolic syndrome: time for a critical appraisal. Diabetes Care.

[8] Eckel RH, Grundy SM, Zimmet PZ (2005). The metabolic syndrome. Lancet.

[9] Kassi E, Pervanidou P, Kaltsas G, Chrousos G (2011). Metabolic syndrome: definitions and controversies. BMC Med.

[10] Hanson RL, Imperatore G, Bennett PH, Knowler WC (2002). Components of the “metabolic syndrome” and incidence of type 2 diabetes. Diabetes.

[11] Laaksonen DE, Lakka HM, Niskanen LK, Kaplan GA, Salonen JT, Lakka TA (2002). Metabolic syndrome and development of diabetes mellitus: application and validation of recently suggested definitions of the metabolic syndrome in a prospective cohort study. Am J Epidemiol.

[12] Lorenzo C, Okoloise M, Williams K, Stern MP, Haffner SM, San Antonio Heart Study (2003). The metabolic syndrome as predictor of type 2 diabetes: the San Antonio heart study. Diabetes Care.

[13] Lakka HM, Laaksonen DE, Lakka TA, Niskanen LK, Kumpusalo E, Tuomilehto J, Salonen JT (2002). The metabolic syndrome and total and cardiovascular disease mortality in middle-aged men. JAMA.

[14] Galassi A, Reynolds K, He J (2006). Metabolic syndrome and risk of cardiovascular disease: a meta-analysis. Am J Med.

[15] Alberti KGMM, Zimmet P, Shaw J (2007). International Diabetes Federation: a consensus on type 2 diabetes prevention. Diabetic Med.

[16] Napoli C, Crudele V, Soricelli A, Al-Omran M, Vitale N, Infante T, Mancini FP (2012). Primary prevention of atherosclerosis. Circulation.

[17] DeFronzo RA, Bonadonna RC, Ferrannini E (1992). Pathogenesis of NIDDM: a balanced overview. Diabetes Care.

[18] DeFronzo RA, Ferrannini E (1991). Insulin resistance. A multifaceted syndrome responsible for NIDDM, obesity, hypertension, dyslipidemia, and atherosclerotic cardiovascular disease. Diabetes Care.

[19] Weyer C, Tataranni PA, Bogardus C, Pratley RE (2001). Insulin resistance and insulin secretory dysfunction are independent predictors of worsening of glucose tolerance during each stage of type 2 diabetes development. Diabetes Care.

[20] Kahn SE (2003). The relative contributions of insulin resistance and beta-cell dysfunction to the pathophysiology of type 2 diabetes. Diabetologia.

[21] Ahren B, Pacini G (2005). Islet adaptation to insulin resistance: mechanisms and implications for intervention. Diabetes Obes Metab.

[22] Kim SH, Reaven GM (2008). Insulin resistance and hyperinsulinemia: you can’t have one without the other. Diabetes Care.

[23] Nathan DM, Davidson MB, DeFronzo RA, Heine RJ, Henry RR, Pratley R, Zinman B (2007). Impaired fasting glucose and impaired glucose tolerance. Diabetes Care.

[24] Abdul-Ghani MA, Jenkinson CP, Richardson DK, Tripathy D, DeFronzo RA (2006). Insulin secretion and action in subjects with impaired fasting glucose and impaired glucose tolerance: results from the veterans administration genetic epidemiology study. Diabetes.

[25] DeFronzo RA, Abdul-Ghani MA (2011). Preservation of beta-cell function: the key to diabetes prevention. J Clin Endocrinol Metab.

[26] McAuley KA, Williams SM, Mann JI, Walker RJ, Lewis-Barned NJ, Temple LA, Duncan AW (2001). Diagnosing insulin resistance in the general population. Diabetes Care.

[27] Ross R (1999). Atherosclerosis–an inflammatory disease. N Engl J Med.

[28] Kannel WB, Wolf PA, Castelli WP, D’Agostino RB (1987). Fibrinogen and risk of cardiovascular disease. The Framingham Study. JAMA.

[29] Festa A, D’Agostino R, Tracy RP, Haffner SM, Insulin Resistance Atherosclerosis Study (2002). Elevated levels of acute-phase proteins and plasminogen activator inhibitor-1 predict the development of type 2 diabetes: the insulin resistance atherosclerosis study. Diabetes.

[30] Kamath S, Lip GYH (2003). Fibrinogen: biochemistry, epidemiology and determinants. QJM.

[31] Cistola DP, Robinson MD (2016). Compact NMR relaxometry of human blood and blood components. Trends Analyt Chem.

[32] Hulley SB, Cummings SR, Browner WS (1988). Designing clinical research: an epidemiologic approach.

[33] Kohn M. Sample size calculators for designing clinical research, UCSF Clinical & Translational Science Institute. http://www.sample-size.net/correlation-sample-size/. Accessed 24 June 2017.

[34] O’Bryant SE, Johnson L, Reisch J, Edwards M, Hall J, Barber R, Devous MDS, Royall D, Singh M (2013). Risk factors for mild cognitive impairment among Mexican Americans. Alzheimers Dement.

[35] Robinson MD, Cistola DP (2014). Nanofluidity of fatty acid hydrocarbon chains as monitored by benchtop time-domain nuclear magnetic resonance. Biochemistry.

[36] Motulsky H (2014). Intuitive biostatistics: a nonmathematical guide to statistical thinking.

[37] Huber PJ, Ronchetti EM (2009). Robust statistics.

[38] Klingenberg CP. Regression. MorphoJ User’s Guide. http://www.flywings.org.uk/MorphoJ_guide/frameset.htm?index.htm. Accessed 22 Jan 2015.

[39] Wilcox RR, Keselman HJ (2012). Modern regression methods that can substantially increase power and provide a more accurate understanding of associations. Eur J Pers.

[40] American Diabetes Association (2017). 2. Classification and diagnosis of diabetes. Diabetes Care.

[41] Huber PJ (1964). Robust estimation of a location parameter. Ann Math Statist.

[42] Taylor EN, Forman JP, Farwell WR (2007). Serum anion gap and blood pressure in the national health and nutrition examination survey. Hypertension.

[43] Farwell WR, Taylor EN (2008). Serum bicarbonate, anion gap and insulin resistance in the National Health and Nutrition Examination Survey. Diabet Med.

[44] Farwell WR, Taylor EN (2010). Serum anion gap, bicarbonate and biomarkers of inflammation in healthy individuals in a national survey. CMAJ.

[45] Lundblad R (2003). Considerations for the use of blood plasma and serum for proteomic analysis. Int J Genomics Proteomics.

[46] Robinson MD (2015). Novel diagnostic and analytical applications of benchtop time-domain NMR.

[47] Parker R. Variable clustering in JMP. https://community.jmp.com/t5/JMP-Blog/Variable-clustering-in-JMP/ba-p/30261. Accessed 19 June 2017.

[48] Jones AG, Hattersley AT (2013). The clinical utility of C-peptide measurement in the care of patients with diabetes. Diabet Med.

[49] Kahn SE, Leonetti DL, Prigeon RL, Boyko EJ, Bergstrom RW, Fujimoto WY (1995). Proinsulin as a marker for the development of NIDDM in Japanese–American men. Diabetes.

[50] Mykkanen L, Haffner SM, Kuusisto J, Pyorala K, Hales CN, Laakso M (1995). Serum proinsulin levels are disproportionately increased in elderly prediabetic subjects. Diabetologia.

[51] Hanley AJ, D’Agostino R, Wagenknecht LE, Saad MF, Savage PJ, Bergman R, Haffner SM, Insluin Resistance Atrherosclerosis Study (2002). Increased proinsulin levels and decreased acute insulin response independently predict the incidence of type 2 diabetes in the insulin resistance atherosclerosis study. Diabetes.

[52] Pfutzner A, Kunt T, Hohberg C, Mondok A, Pahler S, Konrad T, Lubben G, Forst T (2004). Fasting intact proinsulin is a highly specific predictor of insulin resistance in type 2 diabetes. Diabetes Care.

[53] Pfutzner A, Forst T (2011). Elevated intact proinsulin levels are indicative of beta-cell dysfunction, insulin resistance, and cardiovascular risk: impact of the antidiabetic agent pioglitazone. J Diabetes Sci Technol.

[54] Schuhmacher JH, Conrad D, Manke HG, Clorius JH, Matys ER, Hauser H, Zuna I, Maier-Borst W, Hull WE (1990). Investigations concerning the potential for using 1H NMR relaxometry or high-resolution spectroscopy of plasma as a screening test for malignant lung disease. Magn Reson Med.

[55] Engstrom G, Hedblad B, Eriksson KF, Janzon L, Lindgarde F (2005). Complement C3 is a risk factor for the development of diabetes: a population-based cohort study. Diabetes.

[56] Ginsberg HN, Zhang Y, Hernandez-Ono A (2006). Metabolic syndrome: focus on dyslipidemia. Obesity.

[57] Reaven G (2012). Insulin resistance and coronary heart disease in nondiabetic individuals. Arterioscler Thromb Vasc Biol.

[58] Gilbert R, Logan S, Moyer VA, Elliott EJ (2001). Assessing diagnostic and screening tests: part 1. Concepts. West J Med.

[59] Felson DT. Screening for disease. A Boston University School of Public Health Web Module. http://sphweb.bumc.bu.edu/otlt/mph-modules/ep/ep713_screening/EP713_Screening_print.html. Accessed 23 June 2017.

[60] Norton S, Matthews FE, Barnes DE, Yaffe K, Brayne C (2014). Potential for primary prevention of Alzheimer’s disease: an analysis of population-based data. Lancet Neurol.

[61] Biessels GJ (2015). Capitalising on modifiable risk factors for Alzheimer’s disease. Lancet Neurol.

[62] De Felice FG, Ferreira ST (2014). Inflammation, defective insulin signaling, and mitochondrial dysfunction as common molecular denominators connecting type 2 diabetes to Alzheimer disease. Diabetes.

[63] Willette AA, Bendlin BB, Starks EJ (2015). Association of insulin resistance with cerebral glucose uptake in late middle-aged adults at risk for alzheimer disease. JAMA Neurol.

[64] Li TC, Yang CP, Tseng ST, Li CI, Liu CS, Lin WY, Hwang KL, Yang SY, Chiang JH, Lin CC (2017). Visit-to-visit variations in fasting plasma glucose and HbA_1c_ associated with an increased risk of Alzhemier disease: Taiwan diabetes study. Diabetes Care.

[65] Blumich B, Haber-Pohlmeier S, Wasif Z (2014). Compact NMR.

[66] Matthews DR, Hosker JP, Rudenski AS, Naylor BA, Treacher DF, Turner RC (1985). Homeostasis model assessment: insulin resistance and beta-cell function from fasting plasma glucose and insulin concentrations in man. Diabetologia.

[67] Wallace TM, Levy JC, Matthews DR (2004). Use and abuse of HOMA modeling. Diabetes Care.

[68] Duncan MH, Singh BM, Wise PH, Carter G, Alaghband-Zadeh J (1995). A simple measure of insulin resistance. Lancet.

[69] Katz A, Nambi SS, Mather K, Baron AD, Follmann DA, Sullivan G, Quon MJ (2000). Quantitative insulin sensitivity check index: a simple, accurate method for assessing insulin sensitivity in humans. J Clin Endocrinol Metab.

[70] Legro RS, Finegood D, Dunaif A (1998). A fasting glucose to insulin ratio is a useful measure of insulin sensitivity in women with polycystic ovary syndrome. J Clin Endocrinol Metab.

[71] Chung BH, Segrest JP, Ray MJ, Brunzell JD, Hokanson JE, Krauss RM, Beaudrie K, Cone JT (1986). Single vertical spin density gradient ultracentrifugation. Methods Enzymol.

