# Supplementary material for: Water T2 as an early, global and practical biomarker for metabolic syndrome: an observational cross-sectional study
Source: J Transl Med. 2017 Dec 19;15:258. doi: 10.1186/s12967-017-1359-5 (PMC5738216; doi:10.1186/s12967-017-1359-5)
Supplement: Supplementary file 1 — Additional file 1: Table S1. Correlation coefficients for plasma water T2-provides a full set of Pearson, Spearman and Huber correlation coefficients for biomarkers that have a statistically significant correlation with plasma water T2. Table S2. Correlation coefficients for Serum water T2-provides a full set of Pearson, Spearman and Huber correlation coefficients for biomarkers that have a statistically significant correlation with serum water T2. Table S3. Principal components analysis with variable clustering-provides clusters of related variables and most representative variable in each cluster. Table S4. Multiple regression models for plasma water T2-provides parameter details for the best multiple regression models for plasma water T2. Table S5. Multiple regression models for Serum water T2-provides parameter details for the best multiple regression models for serum water T2. [file 12967_2017_1359_MOESM1_ESM.pdf]

Supplementary Table S1: Correlation Coefficients for Plasma Water T_2

Biomarker[1,2,3]	n	r (Pearson)	ρ (Spearman)	M-value (Huber)
**Insulin and Glucose Markers**				
Insulin C-peptide	70	-0.63****	-0.58****	-0.65****
HOMA-IR[4] (insulin c-peptide)	70	-0.62****	-0.58****	-0.64****
McAuley Index[4]	70	+0.61****	+0.62****	+0.64****
Insulin	70	-0.57****	-0.53****	-0.57****
HOMA-IR[4] (insulin)	70	-0.54****	-0.46***	-0.56****
QUICKI[4]	70	+0.56****	+0.53****	+0.60****
FIRI[4]	70	-0.56****	-0.51****	-0.58****
Glucose/Insulin ratio[4]	70	+0.56****	+0.52****	+0.55****
Proinsulin	42	-0.53***	-0.44**	-0.53***
HbA1c	69	-0.47***	-0.50****	-0.54****
Glucose	70	-0.26*	-0.28*	-0.28*
**Protein, Viscosity & Liver Markers**				
Total protein, serum	69	-0.54****	-0.56****	-0.56****
Serum globulins	69	-0.49****	-0.51****	-0.53****
Serum % globulins	69	-0.37**	-0.39***	-0.46****
Serum viscosity	65	-0.35**	-0.32**	-0.30*
Total protein, plasma	41	-0.54***	-0.57****	-0.55***
Plasma globulins	41	-0.67****	-0.68****	-0.66****
Plasma % globulins	41	-0.58****	-0.51***	-0.56***
Plasma viscosity	51	-0.54****	-0.53****	-0.47***
γ-glutamyl transferase (GGT)	69	-0.39***	-0.38**	-0.37**
Alanine aminotransferase (ALT)	52	-0.32*	-0.33*	-0.37*
Serine (Ser)	55	+0.54****	+0.54****	+0.55****
Asparagine (Asn)	55	+0.47***	+0.48***	+0.36**
Glutamine (Gln)	55	+0.34*	+0.34*	+0.28*
Threonine (Thr)	55	+0.30*	+0.29*	+0.29*
β-Alanine	55	+0.35*	+0.33*	+0.27*
**Lipid & Lipoprotein Markers**				
Apolipoprotein B (apo B)	70	-0.53****	-0.56****	-0.55****
Non-HDL-cholesterol	70	-0.53****	-0.55****	-0.52****
LDL-cholesterol (LDL-C)	70	-0.51****	-0.53****	-0.50****

Supplementary Table S1 (continued):

Correlation Coefficients for Plasma Water T_2

Biomarker[1,2]	n	r (Pearson)	ρ (Spearman)	M-value (Huber)
**Lipid & Lipoprotein Markers (con't)**				
Total cholesterol	70	-0.50****	-0.53****	-0.50****
LDL particle number (LDL-P)	70	-0.48****	-0.52****	-0.52****
Triglycerides (TG)	70	-0.47****	-0.48****	-0.54****
VLDL-cholesterol (VLDL-C)	63	-0.40**	-0.40**	-0.44***
Remnant-cholesterol (Rem-C)[5]	63	-0.39**	-0.43***	-0.44***
**Inflammation/Blood Cell Markers**				
White blood cell count (WBC)	69	-0.56****	-0.52****	-0.58****
Neutrophil count	69	-0.49***	-0.43***	-0.41***
Platelet count	69	-0.35**	-0.36**	-0.42***
Lymphocyte count	69	-0.31**	-0.32**	-0.40***
Monocyte count	69	-0.29*	-0.28*	-0.29*
Red cell distribution width (RDW)	69	-0.24*	-0.31**	-0.33**
Mean corpuscular volume (MCV)	69	+0.25*	+0.26*	+0.28*
Mean corpuscular hemoglobin (MCH)	69	+0.25*	+0.24*	+0.26*
C-reactive protein (CRP)	69	-0.49****	-0.47****	-0.51****
Fibrinogen	43	-0.68****	-0.67****	-0.65****
Complement C3c	40	-0.53***	-0.54***	-0.52***
Complement C4c	40	-0.45**	-0.50***	-0.59****
Platelet activator inhibitor-1 (PAI-1)	43	-0.50***	-0.38*	-0.35*
α1-acid glycoprotein	42	-0.39*	-0.38*	-0.38*
Haptoglobin	41	-0.45**	-0.34*	-0.21(ns)
Interleukin-6 (IL-6)	35	-0.39*	-0.43**	-0.33*
**Electrolyte Markers**				
Lactate	41	-0.49**	-0.49**	-0.53***
Anion Gap, uncorrected	69	-0.43***	-0.49****	-0.55****
Anion Gap, corrected for albumin	68	-0.41***	-0.46****	-0.44***
$Cl^- + CO_2$ (HCO_3^-)	69	+0.41***	+0.40***	+0.36**
**Other Markers**				
Serum T_2	67	+0.80****	+0.79****	+0.81****
Plasma T_1	70	+0.46****	+0.54****	+0.55****

Supplementary Table S1 (continued):
Correlation Coefficients for Plasma Water T_2

Footnotes

[1]All blood samples were drawn in the early morning following a 12-hour overnight fast. *p<0.05; **p<0.01; ***p<0.001; ****p<0.0001.

[2]This table includes those biomarkers that were correlated with plasma water T_2. Ambiguous correlations are described in the Methods section.

[3]Many of the variables were natural-log transformed in order to meet the normality condition, an assumption inherent to the Pearson correlation. The correlation coefficients reported here and in Supplementary Tables 1 and 2 are for the ln-transformed variables, except the McAuley Index and QUICKI, as these indices are inherently ln-transformed. Other variables that were normally distributed and analyzed without ln transformation were plasma and serum T_2, total serum and plasma protein, serum and plasma globulins and % globulins, serum and plasma viscosity, HbA1c, LDL-C, LDL-P, total C, apolipoprotein B, lymphocyte and platelet counts, lactate, and complement C3c.

[4]As defined elsewhere: McAuley Index (37), HOMA-IR (61, 63), QUICKI (65), FIRI (64), G/I ratio (66)

[5]Remnant cholesterol is defined as intermediate-density lipoprotein (IDL) plus $VLDL_3$, as determined using the Vertical Autoprofile method (112).

Supplementary Table S2: Correlation Coefficients for *Serum* Water T_2

Biomarker[1,2,3]	n	r (Pearson)	ρ (Spearman)	M-value (Huber)
**Insulin & Glucose Markers**				
McAuley Index[4]	69	+0.58****	+0.61****	+0.70****
Insulin	69	-0.54****	-0.52****	-0.60****
HOMA-IR[4] (insulin)	69	-0.52****	-0.49****	-0.58****
QUICKI[4]	69	+0.55****	+0.52****	+0.57****
FIRI[4]	69	-0.55****	-0.52****	-0.59****
Glucose/Insulin ratio[4]	69	+0.53****	+0.52****	+0.58****
Insulin c-peptide	69	-0.50****	-0.47****	-0.45****
HOMA-IR[4] (c-peptide)	69	-0.50****	-0.47***	-0.46****
Proinsulin	43	-0.55***	-0.49**	-0.60***
HbA1c	68	-0.36***	-0.40****	-0.43****
Glucose	69	-0.28*	-0.28*	-0.28*
**Protein, Viscosity & Liver Markers**				
Total protein, serum	68	-0.76****	-0.78****	-0.79****
Serum globulins	68	-0.59****	-0.63****	-0.65****
Serum % globulins	68	-0.40***	-0.45***	-0.50****
Serum viscosity	67	-0.46****	-0.44***	-0.45***
Total protein, plasma	42	-0.70****	-0.72****	-0.72****
Plasma globulins	42	-0.72****	-0.74****	-0.69****
Plasma % globulins	42	-0.53***	-0.51***	-0.45**
Plasma viscosity	52	-0.50***	-0.56****	-0.56****
Immunoglobulin G (IgG)	44	-0.48***	-0.47**	-0.66****
Alanine aminotransferase (ALT)	50	-0.31*	-0.33*	-0.35**
Tyrosine (Tyr)	54	-0.43**	-0.42**	-0.43***
1-Methyl-Histidine	54	+0.28*	+0.33*	+0.32*
**Lipid & Lipoprotein Markers**				
Apolipoprotein B (apoB)	69	-0.53****	-0.54****	-0.52****
Non-HDL-cholesterol	69	-0.56****	-0.55****	-0.52****
LDL-cholesterol (LDL-C)	69	-0.55****	-0.52****	-0.53****
Total cholesterol	69	-0.54****	-0.51****	-0.51****
LDL particle number (LDL-P)	68	-0.55****	-0.56****	-0.54****
Triglycerides (TG)	69	-0.44***	-0.48****	-0.54****
VLDL-cholesterol (VLDL-C)	62	-0.44***	-0.43***	-0.49****
Remnant-cholesterol (Rem-C)[5]	62	-0.46***	-0.46***	-0.53****
LpPLA2	68	+0.26*	+0.29*	+0.29*

Supplementary Table S2 (continued):

Correlation Coefficients for *Serum* Water T_2

Biomarker[1,2,3]	n	r (Pearson)	ρ (Spearman)	M-value (Huber)
**Inflammation & Blood Cell Markers**				
White blood cell count (WBC)	68	-0.42***	-0.42***	-0.47****
Neutrophil count	68	-0.34**	-0.31*	-0.37**
Lymphocyte count	68	-0.33**	-0.32**	-0.36**
Red blood cell count	68	-0.28*	-0.24*	-0.26*
Hematocrit	68	-0.25*	-0.24 (ns)	-0.26*
C-reactive protein (CRP)	68	-0.25*	-0.29*	-0.31**
Fibrinogen	44	-0.48**	-0.44**	-0.40**
Complement C3c	41	-0.39*	-0.39*	-0.44**
Complement C4c	41	-0.43**	-0.39*	-0.43**
**Electrolyte Markers**				
Lactate	42	-0.47**	-0.50***	-0.49***
Anion Gap, uncorrected	68	-0.41***	-0.44***	-0.43***
Anion Gap, corrected for albumin	67	-0.35**	-0.40***	-0.39***
Cl^- + CO_2 (HCO_3^-)	68	+0.36**	+0.33**	+0.30*
**NMR Markers**				
Plasma T_2	67	+0.80****	+0.79****	+0.81****
Plasma T_1	68	+0.32**	+0.40***	+0.45***
Serum T_1	69	+0.61****	+0.63****	+0.66****

Footnotes

[1]All blood samples were drawn in the early morning following a 12-hour overnight fast. *p<0.05; **p<0.01; ***p<0.001; ****p<0.0001.

[2]This table includes those biomarkers that were correlated with serum water T_2. Ambiguous correlations are described in the Methods section.

[3]Many variables were natural-log transformed in order to meet the normality condition, an assumption inherent to the Pearson correlation. The correlation coefficients reported here and in Supplementary Tables 1 and 2 are for the ln-transformed variables, except the McAuley Index and QUICKI, as these indices are inherently ln-transformed. Other variables that were normally distributed and analyzed without ln transformation were plasma and serum T_2, total serum and plasma protein, serum and plasma globulins and % globulins, serum and plasma viscosity, HbA1c, LDL-C, LDL-P, total C, apolipoprotein B, lymphocyte and platelet counts, lactate, and complement C3c.

Supplementary Table S2 (continued):
Correlation Coefficients for Serum Water T_2

[4]As defined elsewhere: McAuley Index (37), HOMA-IR (61, 63), QUICKI (65), FIRI (64), G/I ratio (66)

[5]Remnant cholesterol is defined as intermediate-density lipoprotein (IDL) plus $VLDL_3$, as determined using the Vertical Autoprofile method (112).

Supplementary Table S3:
Principal Components Analysis with Variable Clustering

Cluster	Most Representative Variable	Other variables in cluster
1	insulin c-peptide	insulin, proinsulin, glucose, ALT, GGT, RDW
2	plasma globulins	serum globulins, serum %globulins, plasma %globulins
3	serum viscosity	plasma viscosity, total plasma protein
4	serum water T_2	serum T_1, total serum protein
5	non-HDL-cholesterol	LDL-C, IDL-C, VLDL-C, total cholesterol, TG, apolipoprotein B, LDL-P, phospholipids
6	white blood cell count	neutrophil & platelet counts, C-reactive protein, erythrocyte sedimentation rate, haptoglobin
7	fibrinogen	complement C3c, C4c, IL-6, Ser, Asn, Thr, plasma water T_2
8	mean corpuscular hemoglobin	lymphocyte count
9	$Cl^- + CO_2^-$	anion gap, anion gap corrected for [albumin]

Supplementary Table S4: Multiple Regression Models for Plasma Water T_2

Model	R^2_{adj} (observations)[1]	Predictor Variables	Coefficients	p values
1	0.67 (68)	ln insulin c-peptide	-48.9 ± 10.1	<0.0001
		total serum protein	-46.6 ± 10.5	<0.0001
		total cholesterol	-0.3 ± 0.1	0.0048
		ln WBC count	-69.4 ± 18.6	<0.0001
		y-intercept	1330.9 ± 74.1	<0.0001
2	0.72 (41)	ln insulin c-peptide	-46.2 ± 14.2	0.0024
		total plasma protein	-40.1 ± 13.0	0.0039
		apolipoprotein B	-0.7 ± 0.2	0.0025
		ln WBC count	-56.8 ± 23.3	0.0200
		y-intercept	1256.8 ± 95.7	<0.0001
3	0.72 (41)	ln insulin c-peptide	-44.7 ± 14.1	0.0031
		plasma globulins	-66.3 ± 15.6	0.0001
		apolipoprotein B	-0.8 ± 0.2	0.0008
		y-intercept	1058.4 ± 45.4	<0.0001
4	0.75 (41)	ln insulin c-peptide	-32.1 ± 14.6	0.0345
		plasma globulins	-57.9 ± 15.4	0.0006
		apolipoprotein B	-0.7 ± 0.2	0.0013
		ln fibrinogen	-55.2 ± 25.0	0.0339
		y-intercept	1330.2 ± 130.6	<0.0001

[1]Models 2, 3 and 4 included predictor variables that were collected during phase 2 only (n=42), while model 1 used only variables that were collected during *both* phase 1 and phase 2 of the study (n=72). R^2_{adj} is the R^2 value adjusted for the number of predictor variables.

Supplementary Table S5: Multiple Regression Models for *Serum* Water T_2

Model	R^2_{adj} (observations)[1]	Predictor Variables	Coefficients	p values
1	0.71 (67)	ln insulin	-16.3 ± 7.7	0.0374
		total serum protein	-89.2 ± 10.5	<0.0001
		LDL-P	-0.04 ± 0.01	0.0002
		y-intercept	1544.2 ± 70.2	<0.0001
2	0.72 (68)	ln insulin c-peptide	-23.6 ± 9.3	0.0137
		total serum protein	-93.2 ± 9.8	<0.0001
		apolipoprotein B	-0.65 ± 0.16	0.0001
		y-intercept	1557.8 ± 68.0	<0.0001
3	0.75 (41)	lactate	-19.2 ± 8.6	0.0309
		total serum protein	-89.9 ± 13.1	<0.0001
		apolipoprotein B	-0.92 ± 0.19	<0.0001
		y-intercept	1584.4 ± 89.2	<0.0001
4	0.76 (41)	McAuley Index	9.6 ± 2.6	0.0007
		total serum protein	-89.3 ± 12.7	<0.0001
		ln fibrinogen	-69.9 ± 20.5	0.0015
		y-intercept	1774.8 ± 164.8	<0.0001

[1]Models 3 and 4 included predictor variables that were collected during phase 2 only (n=42), while models 1 and 2 used variables that were collected during both phase 1 and phase 2 of the study (n=72). R^2_{adj} is the R^2 value adjusted for the number of predictor variables.
